# TRAIP/RNF206 is required for recruitment of RAP80 to sites of DNA damage

**DOI:** 10.1038/ncomms10463

**Published:** 2016-01-19

**Authors:** Nam Soo Lee, Hee Jin Chung, Hyoung-June Kim, Seo Yun Lee, Jae-Hoon Ji, Yoojeong Seo, Seung Hun Han, Minji Choi, Miyong Yun, Seok-Geun Lee, Kyungjae Myung, Yonghwan Kim, Ho Chul Kang, Hongtae Kim

**Affiliations:** 1 Department of Biological Sciences, Sungkyunkwan University, Suwon 440-746, Republic of Korea; 2 Genomic Instability Research Center, Ajou University School of Medicine, Suwon 16499, Korea; 3 Department of Biological Sciences, Sookmyung Women's University, Seoul 04310, Republic of Korea; 4 Department of Science in Korean Medicine, College of Korean Medicine, Kyung Hee University, Seoul 02447, Korea; 5 Center for Genomic Integrity, Institute for Basic Science (IBS), Ulsan National Institute of Science and Technology, Ulsan 44919, Korea; 6 Center for Neuroscience Imaging Research, Institute for Basic Science, Sungkyunkwan University, Suwon 440-746, Korea

## Abstract

RAP80 localizes to sites of DNA insults to enhance the DNA-damage responses. Here we identify TRAIP/RNF206 as a novel RAP80-interacting protein and find that TRAIP is necessary for translocation of RAP80 to DNA lesions. Depletion of TRAIP results in impaired accumulation of RAP80 and functional downstream partners, including BRCA1, at DNA lesions. Conversely, accumulation of TRAIP is normal in RAP80-depleted cells, implying that TRAIP acts upstream of RAP80 recruitment to DNA lesions. TRAIP localizes to sites of DNA damage and cells lacking TRAIP exhibit classical DNA-damage response-defect phenotypes. Biochemical analysis reveals that the N terminus of TRAIP is crucial for RAP80 interaction, while the C terminus of TRAIP is required for TRAIP localization to sites of DNA damage through a direct interaction with RNF20–RNF40. Taken together, our findings demonstrate that the novel RAP80-binding partner TRAIP regulates recruitment of the damage signalling machinery and promotes homologous recombination.

## Abstract (web summary)

Recruiting DNA damage repair factors to the sites of DNA damage is critical for the maintenance of genome integrity. Here the authors identify that the TRAF-interacting protein (TRAIP/RNF206) is required for normal recruitment of RAP80 to DNA lesions and the stimulation of homologous recombination.

## Graphical abstract

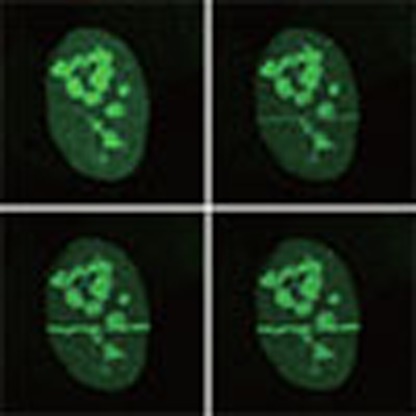

Cellular DNA is under constant threats from multiple sources. Deleterious DNA damage can be caused by exogenous agents, including ultraviolet radiation and ionizing radiation (IR), and can occur as a result of aberrant endogenous events including DNA replication stalling/collapse and mitotic errors^1^,^2^,^3^,^4^. In response to genotoxic stress, cells activate DNA-damage response (DDR) that coordinates multiple intricately controlled pathways, involving damage-sensing, signalling and DNA repair factors, to ensure that these DNA lesions are repaired accurately and genomic integrity is maintained^2^,^3^. DNA double-strand breaks (DSBs) are the most detrimental form of DNA lesions that, if left unrepaired, would lead to genomic instability, such as chromosome translocation, fusion, deletion or mutation^5^,^6^. To protect from these menaces, cells utilize multiple DSB repair pathways at different stages of the cell cycle, including non-homologous end-joining (NHEJ), homologous recombination (HR) and alternatively microhomology-mediated end joining^7^.

On DNA damage, post-translational modifications of histone and repair proteins are induced rapidly as part of the DDR^8^,^9^,^10^,^11^. The activated ataxia-telangiectasia mutated (ATM) immediately phosphorylates H2AX, and the resulting γH2AX bound to DSB ends creates a binding site for the MDC1 protein. The ATM and MRE11 -RAD50 -NBS1 (MRN) complex then become associated with MDC1 to promote the propagation of γH2AX signal at DNA DSB ends. Next, phosphorylated MDC1 recruits additional DNA repair factors, including RNF8/168 E3 ubiquitin ligase, to the sites of DNA damage. The ubiquitination of histone H2A and H2AX by RNF8/168 as part of the DDR is required for the retention of 53BP1 and BRCA1 and provides an additional mark for the recruitment of other DNA repair factors to allow proper DNA repair of the DSBs^12^,^13^. One of these downstream effectors of ubiquitinated histone H2A and H2AX is RAP80 (receptor-associated protein 80). RAP80 mediates DNA DSB repair by recruiting the BRCA1-A complex to DNA-damage sites through CCDC98 (refs 14, 15). Ubiquitin-interacting motif (UIM) of RAP80 specifically recognizes Lys 63-linked histone ubiquitination, H2A and H2AX, at sites of DNA damage^16^,^17^,^18^. The translocation of the BRCA1-A complex to DNA-damage sites has been shown to regulate the G2/M checkpoint and DNA-damage repair and is required for cell survival^19^.

In addition to H2A and H2AX, histone H2B protein has been shown to be ubiquitinated in response to DNA damage. During transcription, histone chaperone FACT is recruited to transcription-blocked sites, and subsequently recruits PAF and H2B monoubiquitin E3 ligase, RNF20–RNF40 complex, to promote displacement of H2A/H2B at the sites^11^. Recently, it has been shown that monoubiquitination of H2B by RNF20 facilitates HR repair^10^. RNF20 is recruited to DNA-damage lesions independently of H2A and H2AX, and is required for the recruitment of chromatin remodeller SNF2h ATPase and DNA repair factors, BRCA1 and RAD51, to regulate the HR pathway^10^. Therefore, histone modification by ubiquitination is important for the regulation of chromatin structure and loading of DNA repair factors, including BRCA1 and 53BP1 that determines the DNA DSB repair pathway choice of either NHEJ or HR.

TRAIP (TRAF (tumour necrosis factor receptor-associated factor)-interacting protein), also known as RNF206, interacts with the TRAF signalling complex and negatively regulates the signalling transduction of TRAF2-mediated nuclear factor-kappa B activation^20^,^21^. TRAIP is essential for embryo development in Drosophila that is encoded by NOPO (no pole)^22^. In addition, TRAIP deficiency in mice results in embryonic lethality^23^. TRAIP protein has a functional E3 ubiquitin ligase RING finger domain at the N-terminal^22^,^24^. However, its substrates in response to DNA damage have not been identified. Here we reveal that, as a novel binding partner for RAP80, TRAIP targets RAP80 to DNA lesions to regulate G2/M DNA-damage checkpoint control and HR.

## Results

### Identification of TRAIP as a novel RAP80-binding protein

**Figure 1:**
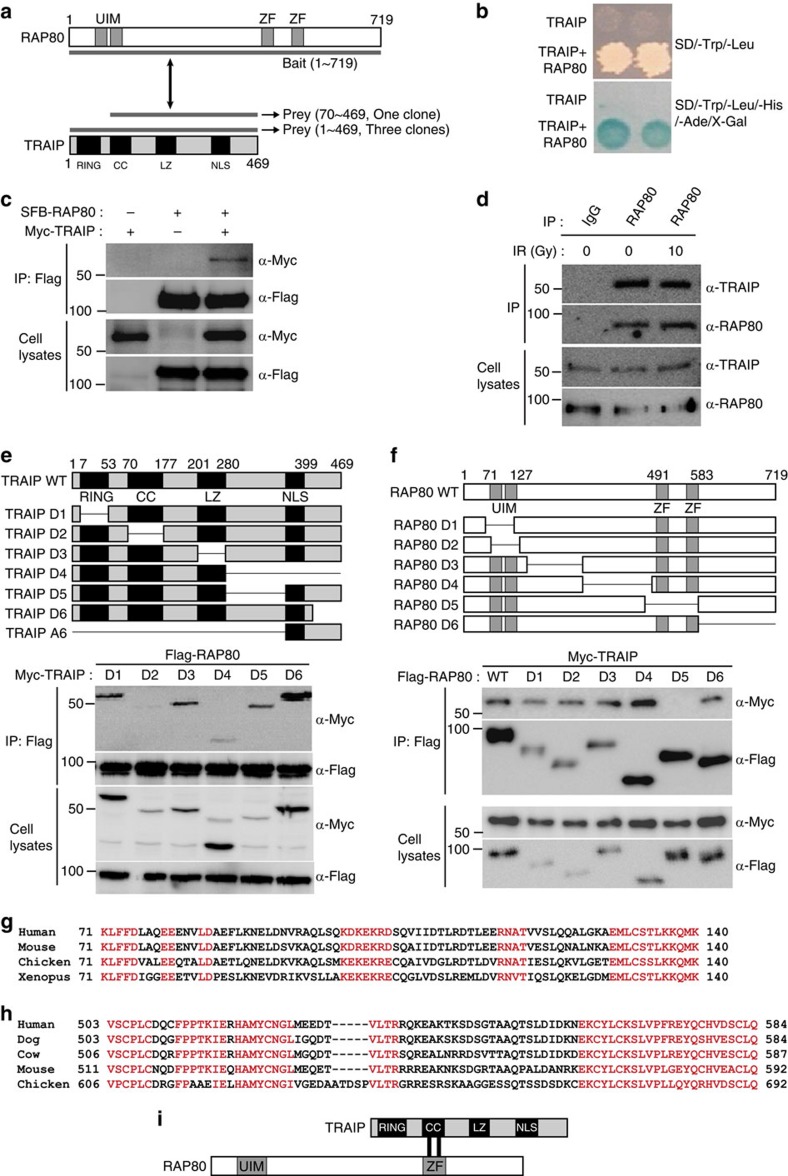
Identification of TRAIP as a novel RAP80-binding protein. (**a**) Schematic structure of RAP80 and TRAIP. The grey line below RAP80 indicates bait, and the two grey lines above RAP80 highlight the prey clones identified in the yeast two-hybrid screen. (**b**) Indicated protein expression vectors were co-transformed into yeast AH109 cells and growing colonies were assessed on high stringent media. Blue colonies on the selective media indicate a positive interaction. (**c**) Cell extracts from 293 T cells expressing SFB-RAP80 and Myc-TRAIP were immunoprecipitated with anti-Flag antibody and then analysed by immunoblotting. (**d**) The interaction between endogenous TRAIP and RAP80. 293 T cells were exposed to 0 or 10 Gy of ionizing radiation and harvested after 1 h. Immunoprecipitation was performed using control IgG or anti-RAP80 antibodies and then analysed by immunoblotting. (**e**,**f**) Upper panel shows diagram of wild-type (WT) TRAIP and internal deletion mutants (**e**), and WT RAP80 and internal deletion mutants (**f**). Numbers indicate amino acids. 293 T cells were co-transfected with plasmids encoding Flag-RAP80 and Myc-tagged TRAIP WT or deletion mutants (**e**), and Myc-TRAIP and Flag-tagged RAP80 WT or deletion mutants (**f**). Cell lysates were subjected to immunoprecipitation with anti-Flag antibody and then analysed by immunoblotting (bottom panel). (**g**,**h**) Amino-acid sequence alignment of the RAP80-binding region of TRAIP (**g**), and the TRAIP-binding region of RAP80 (**h**) in mammalian species. (**i**) Schematic illustration of the binding domain architecture of TRAIP and RAP80. See full blots in the Supplementary Fig. 14.

RAP80 is one of the key molecules in DDR^13^,^14^,^15^,^16^. To gain further insight into the molecular mechanisms of RAP80 in the DDR pathway, we performed yeast two-hybrid screening with the full-length RAP80 as a bait and a human HeLa cDNA library as a prey (Fig. 1a). Among 2 × 10^6^ transformants, 37 positive clones with the highest galactosidase activity were obtained (Supplementary Table 1). The positive clones were analysed by sequencing, and immediately TRAIP caught our attention as four out of thirty-seven positive clones contained full-length (three clones) or N-terminal truncated TRAIP (one clone; Fig. 1a). In addition, the TRAIP is composed of conserved motifs including RING, coiled coil (CC), leucine zipper and nuclear localization signal, all of which are often found in factors implicated in DDR or DNA repairs (Fig. 1a). To validate our initial screening, we performed yeast two-hybrid screening again using RING domain-deleted TRAIP as bait and identified one wild-type (WT) and one N-terminal-deleted RAP80 as positive clones (Supplementary Fig. 1). We further confirmed the potential interaction between RAP80 and TRAIP, with defined yeast two-hybrid analysis (Fig. 1b) and immunoprecipitation in cells overexpressing SFB-RAP80 and Myc-TRAIP (Fig. 1c). To detect endogenous TRAIP, we raised an anti-TRAIP antibody in the laboratory. Western blot analysis demonstrated that the TRAIP antibody detects both endogenous and overexpressed TRAIP (Supplementary Fig. 2A,B), which disappeared when TRAIP is depleted with short interfering RNA (siRNA) against TRAIP (Supplementary Fig. 2C). Using this antibody, we proved for endogenous TRAIP in the endogenous RAP80 immunoprecipitates and noticed that the interaction was not altered in response to DNA damage induced by IR, indicating that the RAP80–TRAIP interaction is not promoted by DNA DSBs (Fig. 1d). To identify critical domains of TRAIP responsible for the RAP80 binding, we generated a series of deletion mutants of Myc-tagged TRAIP (TRAIP-D1 to TRAIP-D6) and co-transfected each of TRAIP deletion mutant with WT Flag-tagged RAP80, followed by co-immunoprecipitation (Fig. 1e, top). Although TRAIP-D1, -D3, -D4, -D5 and -D6 interact with RAP80, the TRAIP-D2 TRAIP mutant failed to do so, indicating that the CC domain (residues 70–177) of TRAIP is critical for its interaction with RAP80 (Fig. 1e, bottom and Fig. 1i). These findings were further supported by the results showing that the GST–TRAIP–CC fusion protein was solely sufficient for binding to RAP80 (Supplementary Fig. 3A). This result prompted us to align the human TRAIP amino-acid sequences with those of TRAIP from other species. It turns out that the CC domain of TRAIP is highly conserved among species, implying that the CC domain is critical for the functions of TRAIP (Fig. 1g). We then constructed a series of internal deletion mutants of RAP80 (RAP80-D1 to RAP80-D6) to determine which regions of RAP80 are important for its association with TRAIP (Fig. 1f, top). Consistent with the yeast two-hybrid assay results (Supplementary Fig. 1), we found that the C-terminal region (residues 491–583) of RAP80 interacted with TRAIP (Fig. 1f, bottom and Fig. 1i). The TRAIP-interacting domain of RAP80 is corresponding to the zinc finger (ZF) motif of RAP80, which is also highly conserved in vertebrates (Fig. 1h). In addition, glutathione *S*-transferase (GST) pulldown experiment proves that the ZF motif of RAP80 alone is sufficient for the interaction with TRAIP (Supplementary Fig. 3B). Taken together, these findings demonstrate that TRAIP is a novel binding partner of RAP80.

### TRAIP travels to DNA lesions via its C-terminal region

**Figure 2:**
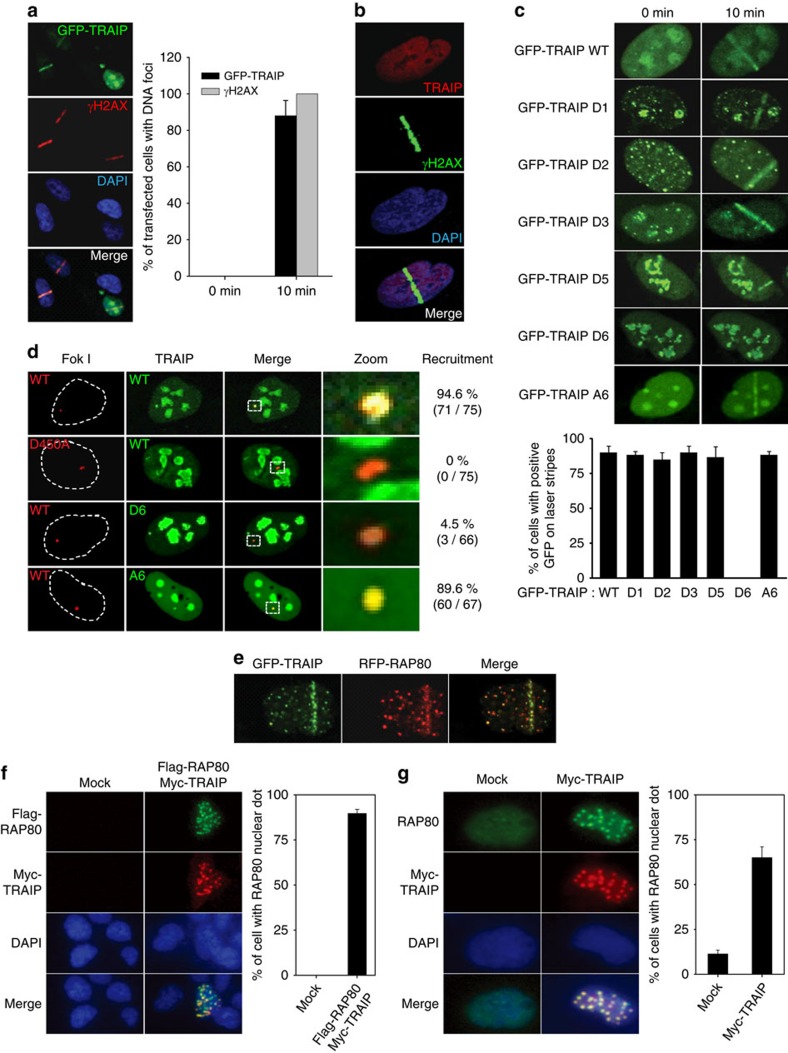
Subcellular translocalization of TRAIP to the DNA-damage site. (**a**) U2OS cells expressing GFP–TRAIP were subjected to laser microirradiation. The laser stripes were examined for 10 min after the microirradiation. The percentage of cells with positive GFP on the laser stripes was determined. More than 50 nuclei per condition. (**b**) U2OS cells were treated with laser microirradiation. After 30 min, the cells were fixed and stained with anti-TRAIP and -γH2AX antibodies. (**c**) U2OS cells expressing indicated proteins were subjected to laser microirradiation (top), and the cells with positive GFP on laser stripes (10 min after laser microirradiation) were presented with bar graph. More than 50 cells in each experiment. (**d**) mCherry-LacI-FokI was co-transfected with indicated GFP-tagged expression vector into U2OS-DSB reporter cells. After 48 h, live cell imaging was performed with confocal microscopy. GFP-positive cells over total counts were denoted. (**e**) U2OS cells expressing GFP–TRAIP and RFP-RAP80 were subjected to laser microirradiation. Colocalization of TRAIP with RAP80 at laser-induced DNA lesions (10 min after laser microirradiation). (**f**) Immunofluorescence assays were performed with 293 T cells expressing Flag-RAP80 and Myc-TRAIP. Cells with TRAIP and RAP80 positive were counted and presented with the bar graph. More than 50 nuclei per condition. (**g**) Overexpressed TRAIP recruits the endogenous RAP80 to the nuclear sparkle. Immunofluorescence assays were performed with 293 T cells expressing Myc-TRAIP using anti-Myc and anti-RAP80 antibodies. Percentage of RAP80-positive cells was determined. More than 50 nuclei per condition. DAPI was used as an indicator for the nucleus. The results represent the average of three independent experiments in each comparison. Error bars indicate the s.d.

The RAP80 translocalizes to sites of DNA lesions^12^,^13^,^14^,^15^,^16^. Since TRAIP interacts with RAP80, we hypothesized that TRAIP might also travel to the sites of DNA damage. To examine whether the TRAIP localizes to sites of DNA damage, we employed laser microirradiation that gives rise to limited amounts of DNA lesions in localized nuclear regions. To visualize the TRAIP, green fluorescence protein (GFP)-tagged TRAIP is constructed and transiently expressed in U2OS cells. On microirradiation, GFP–TRAIP was found to accumulate at the sites of DNA damage (90% in U2OS cells), which is colocalized with γH2AX, the marker for DNA damage (Fig. 2a). Using the TRAIP-specific antibody, we also confirmed that the endogenous TRAIP accumulates at the sites of laser-induced DNA lesions, which is also colocalized with γH2AX (Fig. 2b). We next determined specific regions of TRAIP responsible for its translocation to DNA-damage sites. Cells transiently expressing each of GFP-tagged TRAIP WT, D1, D2, D3, D5, D6 and A6 mutant (Fig. 2c) were irradiated, and cells with positive GFP on laser stripes were counted. We found that more than 80% of cells expressing TRAIP WT, D1, D2, D3 and D5 were positive for GFP on the laser stripes. However, interestingly, the TRAIP-D6 failed to accumulate at the DNA lesions, indicating that the C terminus of TRAIP, which is not responsible for RAP80 interaction, is critical for TRAIP recruitment to the sites of DNA damage (Fig. 2c). To exclude the possibility that altered protein conformational change is attributed to the inability of the TRAIP-D6 mutant to localize to the laser stripe, we tested the recruitment of the C-terminal region of TRAIP (TRAIP A6 amino acids 390–469) to the sites of DNA damage on microirradiation. As expected, the C-terminal region was solely sufficient for localization to laser stripe (Fig. 2c). We further confirmed the recruitment of WT TRAIP, C-terminal deletion (TRAIP-D6), or C-terminal region (TRAIP A6), using a mCherry-LacI-Fok I nuclease fusion protein that creates single DSB in U2OS cells^25^. The Fok I D450A mutant, which is unable to generate the DSB, could not induce the translocation of TRAIP to the DSB site (Fig. 2d). Consistent with our previous data, TRAIP WT and A6 mutant is colocalized with mCherry-Fok I nuclease at the single DSB site (95% for WT and 90% for TRAIP-A6 in U2OS), whereas D6 mutant failed to do so. Taken together, these data demonstrate that TRAIP travels to sites of DNA damage, and that the TRAIP localization is governed by the C terminus, but the N terminus of TRAIP is critical for the TRAIP–RAP80 interaction.

### TRAIP is colocalized with RAP80 in distinct foci

We next tested whether both TRAIP and RAP80 are recruited to the sites of DNA lesions. As expected, recruitment of both GFP–TRAIP and RFP-RAP80 to damage stripes was observed in cells receiving laser irradiation (Fig. 2e). However, interestingly, we noticed that overexpressed Flag-RAP80 and Myc-TRAIP form foci that are colocalized even without DNA damage, implying that TRAIP and TRAIP-binding proteins are located in some other nuclear bodies in the absence of DNA damage (Fig. 2f). This phenomenon allowed us to determine whether TRAIP forms nuclear foci coinciding with RAP80 outside of DNA damage. We transiently transfected with Myc-TRAIP expression vector into 293 T cells and stained the cells with anti-RAP80 or -Myc antibody. The majority of the endogenous RAP80 protein was spread out in the nucleoplasm without forming foci in the absence of exogenous TRAIP, while endogenous RAP80 nuclear foci were increased when TRAIP was overexpressed regardless of irradiation (Fig. 2g). It has been reported that RAP80 is retained in PML nuclear bodies in the absence of DNA damage, and RAP80 might translocalize to the sites of DNA lesions on DNA damage^26^. As TRAIP increased the RAP80 nuclear foci without IR, we examined whether the TRAIP localized to the PML nuclear bodies. As expected, ectopically expressed TRAIP was found as foci in the PML nuclear bodies (Supplementary Fig. 4A). More specifically, most of Myc-TRAIP colocalized with PML I or IV to the PML nuclear body (Supplementary Fig. 4B). These findings suggest that TRAIP and RAP80 are not only recruited to DNA lesions on DNA damage but also retained together in the PML nuclear bodies in the absence of DNA damage.

### TRAIP is an upstream factor for RAP80 translocalization

**Figure 3:**
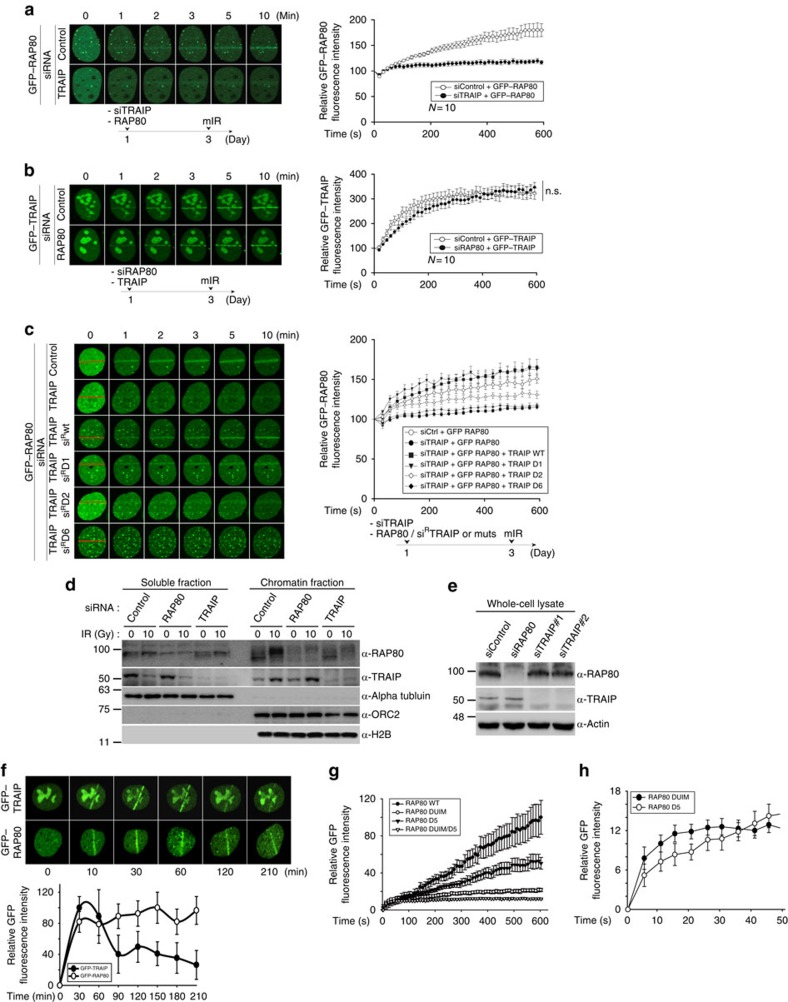
TRAIP is critical for RAP80 recruitment to the sites of DNA lesions. (**a**–**c**) U2OS cells expressing GFP–RAP80 or GFP–TRAIP were transfected with indicated siRNAs or siRNAs together with siRNA-resistant TRAIP WT, TRAIP-D1, -D2 or -D6. After 72 h, cells were subjected to laser microirradiation. Laser stripes were examined at the indicated time points. The intensity of each laser stripe in each time point was determined by averaging values from 10 cells and was graphed in the right panel. Experimental strategy is illustrated. (**d**) Cell fractionation to determine protein localization in response to DNA damage. The 293 T cells were transfected with control, RAP80 or TRAIP siRNA. After 48 h, the transfected cells were exposed to 0 or 10 Gy of ionizing radiation for 1 h. The chromatin fractions were subjected to western blot analysis. (**e**) Western blot analysis for TRAIP or RAP80 protein level in 293 T cells transfected with indicated siRNAs. (**f**) Kinetics of GFP–TRAIP or GFP–RAP80 translocation to DSBs. Average intensity of the laser stripes from 10 cells was presented with graph. (**g**,**h**) GFP-tagged RAP80 WT or indicated mutant was individually transfected into U2OS cells. After 72 h, the cells were subjected to laser microirradiation. The intensity of each laser stripe in each time point was determined by averaging values from 10 cells. Error bars indicate the s.d. See full blots in the Supplementary Fig. 14.

Since TRAIP interacts with RAP80 and also travels to the sites of DNA damage, we asked whether TRAIP regulates RAP80 recruitment to DNA-damage sites or conversely. To evaluate the recruitment of TRAIP and RAP80 at DSBs, we first analysed the kinetics of GFP-fused proteins' accumulation on stripes after laser microirradiation. Surprisingly, we discovered that the accumulation of RAP80 at laser stripes was impaired in cells with siRNA-mediated TRAIP depletion, whereas the accumulation of TRAIP was completely normal in cells with RAP80 depletion (Fig. 3a,b), indicating that TRAIP functions upstream of RAP80 in DDR. The impaired RAP80 recruitment in cells with TRAIP depletion was successfully rescued by expression of siRNA-resistant WT TRAIP and TRAIP-D1 mutant lacking the RING domain of TRAIP. However, co-transfection of siRNA-resistant TRAIP-D2 and -D6 in cells with TRAIP depletion failed to rescue the RAP80 recruitment, indicating that the TRAIP–RAP80 interaction and translocalization of TRAIP to the DNA lesion are critical for RAP80 translocation (Fig. 3c). Since RNF8- and RNF168-mediated DNA-damage-signalling cascade acts upstream of RAP80 recruitment, we tested whether there is a functional connection between TRAIP and RNF8 or RNF168. The IR-induced RNF8 or RNF168 focus formation was merely affected by TRAIP depletion in 293 T cells, and TRAIP accumulation kinetics was normal in U2OS cells with RNF8 depletion (Supplementary Fig. 5A–C), suggesting that TRAIP-dependent RAP80 recruitment to the sites of DNA damage has no functional connection with the canonical RNF8- and RNF168-mediated DDR. In addition, we found that depletion of TRAIP results in severe suppression of IR-induced focus formation of two additional factors, CCDC98 and Merit40, both of which are associated with RAP80 in the BRCA1-A complex (Supplementary Fig. 6A,B), implying that TRAIP regulates the BRCA1-A complex recruitment through direct interaction with RAP80. These findings were further supported by the results, showing that chromatin localization of RAP80 on IR was severely reduced in cells treated with siRNA against TRAIP compared with control siRNA; meanwhile, IR-induced chromatin localization of TRAIP was normal in cells treated with siRNA against RAP80 (Fig. 3d). It is worth noting that the expression level of RAP80 was not altered in cells treated with siRNA against TRAIP-compared mock siRNA, and the level of TRAIP was the same in the presence or absence of RAP80 (Fig. 3e).

### The ZF motif of RAP80 is important for RAP80 translocalization

Next, we investigated how TRAIP regulates the accumulation of RAP80 at the sites of DNA damage. To evaluate kinetics of the recruitment of TRAIP and RAP80 to DNA lesions, we employed a real-time detection method for monitoring protein accumulation at the laser stripes on microirradiation. Each of GFP–TRAIP and GFP–RAP80 expression vector was transfected into U2OS cells, and we were able to detect that both GFP–TRAIP and GFP–RAP80 rapidly translocate to DNA-damage sites within 10 min after laser microirradiation (Fig. 3f). Notably, we observed that the accumulation of GFP–TRAIP at DNA-damage sites peaked at 30–40 min and gradually reduced thereafter, while GFP–RAP80 remained steady up to 210 min (Fig. 3f), implying that the TRAIP plays a key role in early point of RAP80 recruitment, but the RAP80, not TRAIP, might execute the rest of DDR function. These data strongly suggest that, besides UIM recognizing K-63-linked polyubiquitin chain, the ZF motif of RAP80, responsible for TRAIP interaction, is required for its appropriate targeting to DNA lesion. To exploit the coordination of UIM and ZF motif of RAP80 for the recruitment of RAP80 to DNA lesion, we used again the real-time detection method for monitoring protein accumulation at the laser stripes. We observed that the accumulation of TRAIP-interacting motif-deleted mutant, RAP80-D5, was less than WT RAP80, but more than UIM-deleted mutant (RAP80 DUIM; Fig. 3g and Supplementary Fig. 7). However, surprisingly, in the very early time points of the protein assembly at laser stripes, RAP80 D5 recruitment was slower than the RAP80 DUIM mutant, which was reversed after 40 s (Fig. 3h), implying that TRAIP regulates the initial recruitment of RAP80, which is dependent on the ZF motif of RAP80. Consistently, the GFP–RAP80 DUIM/D5 mutant was merely found on the laser stripes, indicating that both UIM and ZF domains of RAP80 are necessary for the recruitment of RAP80 to the sites of DNA damage.

### TRAIP promotes DDR and repair

**Figure 4:**
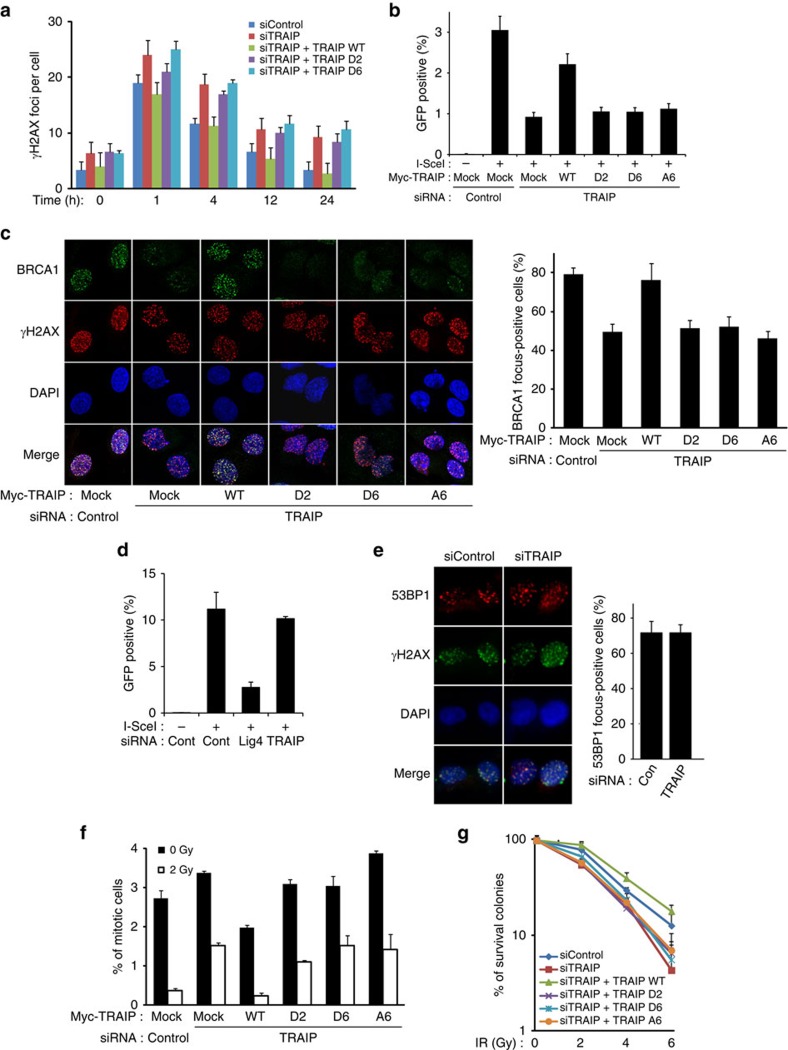
TRAIP is required for DNA-damage checkpoint and homologous recombination repair. (**a**) Counts of γH2AX foci at various time points after 1-Gy ionizing radiation in HeLa cells transfected with indicated siRNAs or combination of siRNA and siRNA-resistant WT TRAIP, TRAIP-D2 or -D6 expression vector. (**b**) Comparison of homologous recombination repair capacity in DR-GFP U2OS cells transfected with indicated siRNAs or combination of siRNA and siRNA-resistant WT TRAIP, TRAIP-D2, -D6 or -A6. (**c**,**e**) 293T cells were transfected with indicated siRNA or combination of siRNA and siRNA-resistant WT TRAIP, TRAIP-D2, -D6 or -A6. After 48 h, transfected 293 T cells were exposed to 10-Gy of ionizing radiation, followed by staining with anti-BRCA1 (**c**), 53BP1 (**e**) or γ-H2AX antibodies. DAPI was used as an indicator for the nucleus. The percentage of cells with positive BRCA1 or 53BP1 foci from 100 counts was determined. (**d**) Non-homologous end joining repair assay was carried out in triplicates using U2OS cells harbouring NHEJ reporter (EJ5-GFP). The reporter cells were transfected with indicated siRNA and the percentage of GFP-positive cells were analysed using flow cytometry. (**f**) G2/M checkpoint in HeLa cells transfected with siRNAs or combination of siRNA and siRNA-resistant WT TRAIP, TRAIP-D2, -D6 or -A6. The transfected cells were exposed to 0 or 2 Gy of ionizing radiation and subjected to staining with antibody to phosphorylated histone H3 and propidium iodide. The percentages of mitotic cells were determined using flow cytometry. (**g**) Radiation sensitivity in HeLa cells transfected with siRNAs or combination of siRNA and siRNA-resistant WT TRAIP, TRAIP-D2, -D6 or -A6. These experiments were performed in triplicate, and the results represent the average of three independent experiments in each comparison. Error bars indicate the s.d.

Given the reported functions of RAP80 in DDRs together with our findings described above, we examined the possible scenario of TRAIP in the DDR pathways, especially DNA-damage repair, checkpoint and IR sensitivity. Indeed, the γH2AX foci slowly disappeared in HeLa cells treated with TRAIP siRNA compared with control siRNA on IR (Fig. 4a and Supplementary Fig. 8A). This phenomenon was fully rescued by expression of siRNA-resistant WT TRAIP, but not by TRAIP-D2 and -D6 (Fig. 4a), demonstrating that the TRAIP–RAP80 interaction and the ability of TRAIP translocalization to the sites of DNA damage is important for IR-induced DNA lesion. Next, using the DR-GFP reporter system, we tested whether depletion of TRAIP leads to HR defects. We found that the efficiency of HR repair was reduced in TRAIP-depleted cells (Fig. 4b and Supplementary Fig. 9A). To remove the off-targeting effects of RNA interference (RNAi)-mediated knockdown of TRAIP, restoration experiments were conducted using the siRNA-resistant TRAIP WT expression vector. The reconstitution in TRAIP-knockdown cells with TRAIP WT successfully rescued HR repair efficiency, but TRAIP-D2, -D6 and -A6 failed to do so, indicating that the TRAIP–RAP80 interaction and the C terminus of TRAIP, which is responsible for TRAIP recruitment to DNA lesion, are critical for promoting HR (Fig. 4b and Supplementary Fig. 9A). These findings were further supported by the facts that IR-induced BRCA1 recruitment to the sites of DNA damage was severely impaired in the cells treated with siTRAIP (Fig. 4c). The impaired IR-induced BRCA1 focus formation was rescued by siRNA-resistant WT TRAIP expression, but not by TRAIP-D2, -D6 and -A6 expression, demonstrating that TRAIP is an upstream factor for RAP80-dependent IR-induced BRCA1 focus formation (Fig. 4c). However, we noticed that non-homologous end joining repair and 53BP1 focus formation are merely affected by the depletion of TRAIP (Fig. 4d,e and Supplementary Fig. 9B), suggesting that TRAIP is not implicated in non-homologous end joining. It has been reported that inactivation of RAP80 and BRCA1 results in defective G2/M checkpoint control. We tested whether the loss of TRAIP would lead to similar defects in the DDR. To this end, we determined mitotic cell population on IR, and found that TRAIP-knockdown cells were unable to arrest in G2 after IR, which was rescued by expression of WT siRNA-resistant TRAIP, but not by TRAIP-D2, -D6 and -A6 (Fig. 4f and Supplementary Figs 8B and 9C). Similarly, TRAIP-knockdown cells displayed enhanced sensitivity to IR (Fig. 4g and Supplementary Figs 8C and 9D), and the reconstitution in TRAIP-knockdown cells with siRNA-resistant TRAIP WT rescued the sensitivity to irradiation, while the TRAIP-D2, -D6 and -A6 did not (Fig. 4g and Supplementary Fig. 9D). These data strongly suggest that TRAIP could be an additive factor for the RAP80 and BRCA1 axes in DDR and homology-directed DSB repair. To verify this idea, we performed genetic interaction analysis between TRAIP and BRCA1 or RAP80 using systematic siRNA-mediated individual and pairwise gene depletion. We found that individual or dual knockdown of both TRAIP and BRCA1 results in a similar level of detrimental effects on IR-induced γH2AX focus formation, HR repair, checkpoint and IR sensitivity, indicating that TRAIP is epistatic to BRCA1 in DDR and repair (Supplementary Fig. 10A–E). The same epistatic analysis revealed that TRAIP is also epistatic to RAP80 in IR-induced γH2AX focus formation, checkpoint and IR sensitivity (Supplementary Fig. 10). The molecular functions of RAP80 in HR repair are still controversial, as some reports demonstrate that RAP80 suppresses HR repair^27^,^28^; however, there are other reports showing that depletion of RAP80 reduces HR frequency^19^,^29^. We found that depletion of RAP80 slightly reduces the HR frequency, but dual depletion of RAP80 and TRAIP showed enhanced defects in HR repair comparable to TRAIP depletion (Supplementary Fig. 10C). Taken together, these data demonstrated that TRAIP is implicated in HR and G2/M checkpoint control and that it plays an important role in the regulation of RAP80 and functional downstream factors including BRCA1.

### RNF20–RNF40 regulates translocation of TRAIP to DSBs

**Figure 5:**
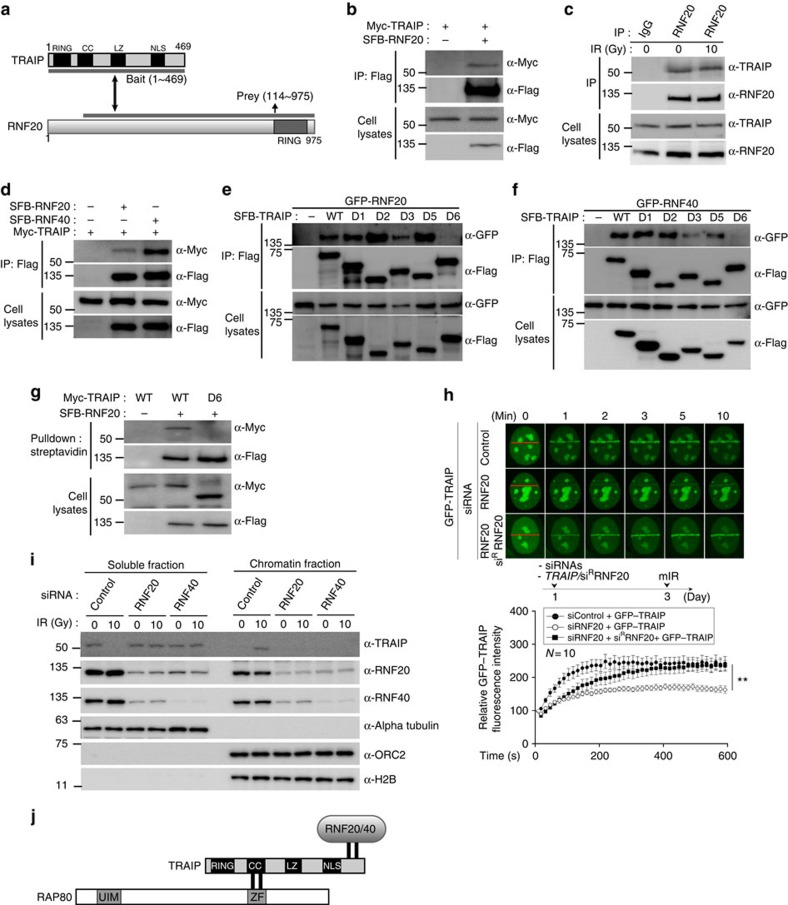
RNF20–RNF40 regulates translocation of TRAIP to DSBs. (**a**) Schematic structure of TRAIP and RNF20. The grey line below TRAIP indicates bait, and the grey line above TRAIP highlights the prey clone identified in the yeast two-hybrid screen. (**b**) Cell extracts from 293 T cells expressing Myc-TRAIP and SFB-RNF20 were immunoprecipitated with anti-Flag antibody and then analysed using immunoblotting. (**c**) The interaction between endogenous TRAIP and RNF20. 293 T cells were exposed to 0 or 10 Gy of ionizing radiation and harvested after 1 h. Immunoprecipitation was performed using control IgG or anti-RNF20 antibodies and then analysed using immunoblotting. (**d**) Cell extracts from 293 T cells expressing indicated proteins were immunoprecipitated with anti-Flag antibody and then analysed by immunoblotting. (**e**,**f**) Domains of TRAIP required for TRAIP–RNF20 or TRAIP-RNF40 interactions. 293 T cells were transfected with plasmids encoding GFP-RNF20 or GFP-RNF40 together with WT TRAIP or serial deletion mutants. Cell lysates were subjected to immunoprecipitation with anti-FLAG antibody and then analysed by immunoblotting. (**g**) 293 T cells were transfected with plasmids encoding SFB-RNF20 with Myc-TRAIP WT or D6 mutant. After 24 h, cell lysates were subjected to pulldown with streptavidin beads and immunoblotted with the indicated antibodies. (**h**) U2OS cells expressing GFP–TRAIP were transfected with siRNF20 or siRNF20 and siRNA-resistant RNF20 expression vector. After 72 h, cells were subjected to laser microirradiation. Laser stripes were examined at the indicated time points. The intensity of each laser stripe in each time point was determined by averaging values from 10 cells and graphed in the bottom panel. Experimental strategy is illustrated. Error bars indicate the s.d. (**i**) The 293 T cells were transfected with control, RNF20 or RNF40 siRNA. After 48 h, the transfected cells were exposed to 0 or 10 Gy of ionizing radiation for 1 h. The chromatin fractions were subjected to western blot analysis. (**j**) Schematic illustration of the binding domain architecture of RNF20/40, TRAIP and RAP80. See full blots in the Supplementary Fig. 14.

Our findings described above suggest that TRAIP acts upstream of RAP80 recruitment to the sites of DNA damage. Then, the next question to be addressed is the underlying molecular mechanism of TRAIP recruitment to DNA lesions. In an attempt to clarify the mechanism, we performed second yeast two-hybrid screening with the full-length TRAIP as bait and a human HeLa cDNA library as prey (Fig. 5a). Among 2 × 10^6^ transformants, a number of positive clones with the highest β-galactosidase activity was isolated. Sequencing analysis revealed two clones from HeLa library encoding N-terminal-deleted RNF20 as positive clones (Fig. 5a). To validate the association between RNF20 and TRAIP, we performed immunoprecipitation assay with the cell lysates transfected with Myc-TRAIP and SFB-RNF20 expression vectors (Fig. 5b) and found that TRAIP interacts with RNF20. We further confirmed that endogenous RNF20 also specifically binds to endogenous TRAIP, and the TRAIP–RNF20 interaction was not promoted in response to DNA damage induced by IR (Fig. 5c). As RNF20 and RNF40 form a heterodimer and stabilize each other^9^,^30^,^31^ (Supplementary Fig. 11), we tested whether TRAIP is co-immunoprecipitated with RNF40. As expected, Myc-TRAIP was detected in the immunoblot with eluates from the immunoprecipitation with anti-Flag antibody (Fig. 5d). To determine the TRAIP domain responsible for its interaction with RNF20 and RNF40, cell lysates from cells expressing each of TRAIP mutants (Fig. 1e) were subjected to immunoprecipitation with anti-Flag antibody and the eluates were immunoblotted with anti-GFP antibody (Fig. 5e,f,j). Interestingly, we found that TRAIP-D6 mutant, the domain of which is critical for TRAIP translocalization to the sites of DNA damage, fails to interact with both RNF20 and RNF40 (Fig. 5e,f). Consistently, TRAIP was detected in RNF20-immunoprecipitated proteins by the immunoblot, but TRAIP-D6 was not detected (Fig. 5g). On the basis of these data we hypothesized that the translocalization of TRAIP might be regulated by the RNF20–RNF40 complex, which is an E3 ubiquitin ligase for H2B at the sites of DNA damage^10^. To test the hypothesis, we measured the assembly of GFP–TRAIP on the laser stripes in cells receiving siRNA against RNF20. Notably, we discovered that the accumulation of TRAIP at DNA-damage sites was reduced in RNF20-depleted cells, which was rescued by expression of siRNA-resistant WT RNF20 (Fig. 5h). The defect of TRAIP recruitment was further confirmed by cell fractionation and western blot analysis. Chromatin localization of TRAIP was significantly reduced in RNF20 or RNF40 siRNA-transfected cells on IR (Fig. 5i). The expression level of TRAIP was the same with or without RNF20 or RNF40 knockdown, and TRAIP knockdown also does not change the expression of RNF20 or RNF40 (Supplementary Fig. 11). The findings described above prompt us to test whether the RNF20–RNF40 heterodimer is implicated in RAP80–BRCA1-dependent DDR. As expected, we found that depletion of RNF20 leads to checkpoint defect, enhanced IR sensitivity and reduced HR repair (Supplementary Fig. 12A–C). Furthermore, IR-induced RAP80 and BRCA1 focus formation was impaired in the 293 T cells with RNF20 depletion (Supplementary Fig. 12D,E). However, consistent with the case in cells with TRAIP depletion, IR-induced 53BP1 focus formation was merely affected by siRNA-mediated RNF20 knockdown (Supplementary Fig. 12F). Taken together, these data suggest that the recruitment of TRAIP to the sites of DNA damage might be regulated by the RNF20–RNF40 complex, which then might take the RAP80–BRCA1 complex to the sites of DNA lesions.

### Reduced expression of TRAIP in human lung cancer tissues

**Figure 6:**
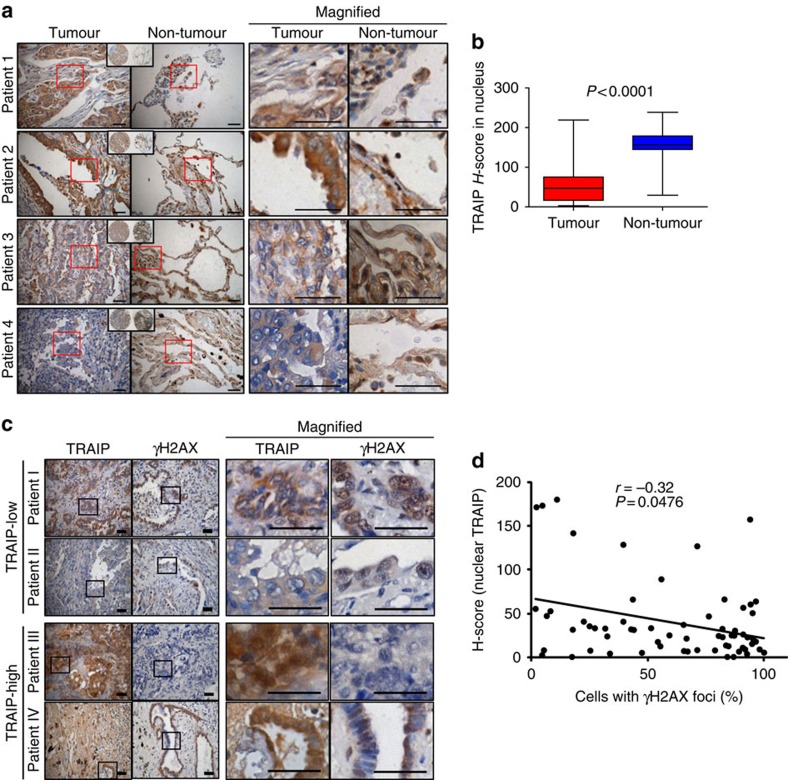
Reduced expression level of TRAIP in human lung adenocarcinoma. (**a**) Immunohistochemical staining of TRAIP in human lung cancer patient tissues and matched normal adjacent tissues. Red squares indicate the magnified region shown to the right panel. (**b**) Box-and-Whisker plots of nuclear TRAIP *H*-scores in lung adenocarcinomas and matched normal adjacent tissues. *P*<0.0001 (*n*=72 for tumour; *n*=71 for normal adjacent tissues). (**c**) Immunohistochemical staining of TRAIP and γH2AX in human lung cancer patient tissues. Enhanced γH2AX foci in the patient tissues with lower nuclear expression of TRAIP (upper panel) and few γH2AX foci in the tissues with higher nuclear expression of TRAIP (lower panel). Black squares indicate the magnified region shown to right panel. Scale bar, 50 μm. (**d**) Pearson correlation coefficient between nuclear TRAIP expression and percentage of cells with γH2AX foci. Graph presents the scatter plot of *H*-score of nuclear TRAIP (*y* axis) and cell percentage with γH2AX foci (*x* axis). *P*<0.05 (*n*=64).

Considering that the interaction between TRAIP and RAP80 is critical for the maintenance of genomic instability by controlling DDR and HR, the loss or downregulation of the expression of either of these genes may be associated with pathophysiological symptoms of, for example, cancer. To support this idea, we verified TRAIP expression in human lung adenocarcinoma patient tissues and matched normal adjacent tissues (≥2 cm away from cancer) using a tissue microarray. The specificity of the TRAIP antibody for immunofluorescent assay was verified (Supplementary Fig. 13). We found that cytoplasmic expression levels of TRAIP between tumour and normal tissues did not show any significant difference. However, nuclear expression of TRAIP in tumour tissues was markedly reduced compared with each matched normal adjacent tissue (Fig. 6a). Nuclear TRAIP expression in 75% cancer tissues was at least twofold lower than that in each corresponding matched normal tissue. *H*-scoring in every sample indicated that nuclear expression of TRAIP greatly decreased in human lung cancer patient tissues (median *H*-score, 46.9) compared with that in each matched normal adjacent tissues (median *H*-score, 156.6; Fig. 6b). Furthermore, we observed an interesting inverse correlation between nuclear expression of TRAIP and γH2AX foci in these tumours (Fig. 6c,d). Increased levels of γH2AX foci were found in the nucleus of the tumours with lower nuclear expression of TRAIP while few γH2AX foci in the patient tissues with higher expression of TRAIP (Fig. 6c). A scatter plot and Pearson correlation coefficient analysis revealed a significant negative correlation (*r*=−0.32) between nuclear expression of TRAIP and γH2AX foci (Fig. 6d). Together, these results suggest that low level of nuclear TRAIP in human lung cancer might be involved in incidence of genome instability—the hallmarks of cancer.

## Discussion

In this study, we identified the TRAIP–RNF206 as a novel binding partner of RAP80 and RNF20–RNF40 using a yeast two-hybrid system. Our results demonstrate that TRAIP, an upstream regulator for RAP80, play a key role in the recruitment of RAP80 to DNA lesions in a coordinative manner, with an already-known K-63-lined polyubiquitin recognition at the sites of DNA damage. TRAIP seems to be retained in the PML nuclear bodies together with RAP80 in the absence of DNA damage (Supplementary Fig. 4). Once DNA damage occurs, TRAIP might take the RAP80 to the sites of DNA damage by the guidance of the RNF20–RNF40 complex, which plays a key role in H2B ubiquitination at the sites of DNA lesions.

Protein–protein interaction and post-translational modification play key roles in the DDR pathway. RAP80, one of the essential proteins in this pathway, is translocated to sites of DNA damage through recognizing of lysine 63-linked ubiquitination of H2A and H2AX by RNF8/168 E3 ligase, and recruits the BRCA1 complex to DNA-damage lesions to enhance HRR through functional interaction of repair factors^9^,^10^,^11^,^12^,^13^. Furthermore, H2B ubiquitination, which is a target of the RNF20–RNF40 complex, is required for recruiting DDR factors to DSBs to promote HR repair through chromatin relaxation in DDR^10^,^11^. However, whether the two different pathways were regulated at the same time or was independent was unclear. Chromatin remodelling and histone modification quickly occur in DDR. The RNF20–RNF40 complex is a well-known E3 ubiquitin ligase for H2B monoubiquitination in transcription and DDR for chromatin relaxation^10^,^11^. Moyal *et al.* suggested that the RNF20–RNF40 complex is involved in chromatin relaxation in DDR^9^. When the RNF20–RNF40 complex is depleted, recruitment of HR and NHEJ factors is reduced. However, on the depletion of TRAIP, recruitment of the HR factors, BRCA1 is reduced, but recruitment of the NHEJ factor, 53BP1, is not. These findings indicate that TRAIP is recruited at DNA-damage lesions in RNF20–RNF40 dependency. TRAIP might coordinately function together with RNF20–RNF40 for the H2B monoubiquitination at the sites of DNA damage as TRAIP contains RING domain, although further studies are required to clarify the issue. Our data demonstrate that TRAIP has two separate domains, one is responsible for its interaction with RAP80 through the N terminus, and the other (C terminus) is critical for TRAIP's localization to the sites of DNA damage. Coincidently, we found that the responsible domain of TRAIP for the interaction with the RNF20–RNF40 complex is the same as the one that is critical for the localization of TRAIP to the DNA lesions (Fig. 5j). Furthermore, recruitment of TRAIP to the microirradiation-induced DNA lesions is severely impaired in the cells treated with siRNA against RNF20. Taken together, possible mechanism for the TRAIP translocalization might be guided by the RNF20–RNF40 complex.

TRAIP might be the responsible factor that takes RAP80 from PML to damage lesions. As a result, TRAIP leads to recruitment of the BRCA1 complex to adjacent DNA-damage lesions by interacting with RAP80 to facilitate DNA-damage checkpoint and HR repair. Since RNF8/168 is not regulated by depletion of TRAIP, localization of 53BP1 is normally observed at DSBs. However, in the absence of TRAIP, RAP80 is not translocated to DSBs; thus, downstream targets of RAP80 and BRCA1 are compromised to be retained at the damage lesions. It seems like localization of RAP80 in PML nuclear bodies is dependent on TRAIP even without DNA damage, suggesting that TRAIP may control the function of RAP80 in PML nuclear bodies. There is evidence that RAP80 binds to ubiquitinated BLM localized at PML nuclear bodies^26^. Furthermore, ATM, BRCA1, Mre11, HIPK2, Ubc9 and p53, which are involved in the DDR pathway, are also localized at PML nuclear bodies^32^. Whether the proteins in PML nuclear bodies are also regulated by TRAIP-dependent ubiquitination warrants study.

Our findings suggest that TRAIP translocates RAP80 from PML nuclear bodies to the sites of DNA damage through interaction with the CC domain of TRAIP and C-terminal region ZF of RAP80. RAP80 translocates to DNA-damage lesions by the UIMs in the N terminus of RAP80 on DNA damage^13^,^14^,^15^,^16^. However, C-terminal-deleted RAP80-mutant D5 is inefficient in accumulating at sites of DNA damage, and RAP80 is also slowly translocated to DNA lesions when TRAIP is downregulated, suggesting that the TRAIP-interacting domain of RAP80 and UIMs of RAP80 are essential for adjusting recruitment/accumulation in response to DNA damage.

Reduced expression level of TRAIP is associated with a human lung adenocarcinoma. Together with our findings in DDR and HR, the clinical relevance of TRAIP emphasizes the important physiological roles of TRAIP, as well.

## Methods

### Plasmids

The SFB-RAP80, RAP80 D-1, D-2, D-3, D-4, D-5 and D-6 deletion mutant expression plasmids were previously described^14^,^16^. GFP-tagged RAP80, RAP80 DUIM, D5 and D5/DUIM expression plasmids were cloned into GFP-tagged mammalian expression vector. Red fluorescent protein (RFP)-tagged RAP80 expression plasmid was cloned into RFP-tagged mammalian expression vector. TRAIP gene was purchased from the Korea Human Gene Bank. The Myc-tagged TRAIP expression plasmid was cloned into Myc-tagged mammalian expression vector, and GFP-tagged TRAIP, D6 and A6 expression plasmids were cloned into GFP-tagged mammalian expression vector. TRAIP D-1, D-2, D-3, D-4, D-5 and D-6 deletion mutants were generated by mutagenesis using a Myc-tagged TRAIP expression plasmid. PML expression plasmids were cloned into Flag-tagged mammalian expression vector.

### Yeast two-hybrid screening

The cDNA of RAP80 or RING domain-deleted TRAIP was subcloned into pGBKT7 as the bait. The two-hybrid screening followed the manufacturer's instruction (Clontech). A HeLa cell cDNA library in pACT2 was used as the prey to screen ≈2 × 10^6^ colonies. The bait and the library DNAs were co-transformed into AH109 yeast strain using the lithium acetate method. The transformants were selected for growth on the Leu^−^, Trp^−^, His^−^ and Ade^−^ solid media containing 30 mM 3-aminotriazole. The β-galactosidase assay was performed by four incubating freeze-fractured colonies on nitrocellulose in Z-buffer (60 mM Na_2_HPO_4_, 40 mM NaH_2_PO_4_, 10 mM KCl, 1 mM MgSO_4_, 0.03 mM β-mercaptoethanol and 2.5 M X-gal) at 30 °C for 30 min.

### Cell culture

U2OS, HeLa, H1299 and human embryonic kidney 293T cell lines were purchased from American Type Culture Collection (Manassas, VA). HeLa and 293T cell lines were maintained in DMEM (Invitrogen, Carlsbad, CA) supplemented with 10% fetal bovine serum (Gibco, Franklin Lakes, NJ) and 1% penicillin/streptomycin (Gibco) at 37 °C in 5% v/v CO_2_.

### siRNAs

Control and RAP80 siRNAs were previously described^14^,^16^. siTRAIP #1: 5′-GCAGCAGGAUGAGACCAAAUU-3′, siTRAIP #2: 5′-GCAAGUUGCAGACAGUCUAUU-3′, siRAP80: 5′-GAAGGAUGUGGAAACUACCUU-3′, siRNF20: 5′-GCUAAAAGAGUCAGAAAAAUU-3′, siRNF40: 5′-GAUGCCAACUUUAAGCUAAUU-3′, siBRCA1: 5′-UCACAGUGUCCUUUAUGUAUU-3′, siRNF8; 5′-GAGAAGCUUACAGAUGUUUUU-3′. siRNAs were transfected into cells using Lipofectamine RNAiMAX reagnent (Invitrogen).

### Antibodies

Anti-RAP80 and -γH2AX antibodies were previously described^14^,^16^. Anti-TRAIP antibody was raised by immunizing rabbits with GST–TRAIP fusion protein. Anti-TRAIP (PA5-27699, Thermo Fisher Scientific) was used for immunohistochemistry. Rabbit RAP80 and TRAIP polyclonal antibody were affinity-purified using the Sulfolink Plus Immobilization and Purification Kit (Pierce, Rockford, IL). Anti-Flag (F3165) and -Actin (A5316) antibodies were purchased from Sigma (St Louis, MO). Anti-Myc antibody (11814150001) was purchased from Roche (Basel, Switzerland). Anti-Tubulin (05-829) and -H2B (07-371) antibodies were purchased from Millipore. Anti-BRCA1 (6954) and -PML (966) antibodies were purchased from Santa Cruz. Anti-53BP1 (4937), -RNF40 (12187) and -phospho H3 (9701) antibodies were purchased from Cell Signaling. Anti-ORC2 antibody (559266) was purchased from BD Science. Anti-RNF20 antibody (33500) was purchased from Abcam. Anti-GFP antibody (632381) was purchased from Clontech. The dilutions of the various antibodies for western blot analysis were as follows: anti-RAP80, 1:1,000; anti-TRAIP, 1:400; anti-FLAG, 1:2,000; anti-Myc, 1:2,000; anti-Tubulin, 1:5,000; anti-ORC2, 1:2,000; anti-H2B, 1:2,000; anti-Actin, 1:5,000; anti-BRCA1, 1:400; anti-RNF20, 1:2,000; anti-RNF40, 1:2,000; and anti-GFP, 1:2,000. The dilutions of the various antibodies for immunofluorescence were as follows: anti-TRAIP, 1:400; anti-RAP80, 1:400; anti-FLAG, 1:200; anti-Myc, 1:200; anti-BRCA1, 1:200; anti-53BP1, 1:200; anti-PML, 1:100; and anti-γ-H2AX, 1:100.

### Transfection and immunoprecipitation

Transient transfection was performed using poly(ethylenimine). For immunoprecipitation, cells were washed with ice-cold PBS and then lysed in NETN buffer (0.5% Nonidet P-40, 20 mM Tris (pH 8.0), 50 mM NaCl, 50 mM NaF, 100 μM Na_3_VO_4_, 1 mM dithiothreitol and 50 μg ml^−1^ phenylmethylsulfonyl fluoride) at 4 °C for 10 min. Crude lysates were cleared by centrifugation at 14,000 r.p.m. at 4 °C for 5 min, and supernatants were incubated with protein A-agarose-conjugated primary antibodies. The immunocomplexes were washed three times with NETN buffer and subjected to SDS–PAGE. Western blotting was performed using the antibodies indicated in the figure legends. Uncropped blots are provided in the Supplementary Fig. 14.

### G2/M cell cycle checkpoint assay

HeLa cells in a 100-mm-diameter plate were transfected twice with indicated siRNAs at 24-h intervals. Forty-eight hours after the second transfection, transfected cells were mock-treated or irradiated at the indicated doses using a radiation source. One hour after irradiation, cells were fixed with 70% (v/v) ethanol at –20 °C for 24 h, and then stained with rabbit antibody to pH3 and incubated with fluorescein isothiocyanate-conjugated goat secondary antibody to rabbit immunoglobulin. The stained cells were treated with RNase A, incubated with propidium iodide and analysed using flow cytometry. Reconstitution assay was carried out in HeLa cells transfected with siTRAIP together with siRNA-resistant WT TRAIP, TRAIP-D2, -D6 or -A6 expression vector.

### Laser microirradiation and imaging of cells

Single strand breaks or DSBs of DNA were induced using a Nikon A1 laser microdissection system (Nikon). U2OS cells in four-well plates were transfected with indicated GFP-tagged expression vector for 48 h and then were incubated with 10 μM of 5-brome-2′-deoxyuridine for 24 h before laser-induced DSBs. Ten cells per well were subjected to laser-induced DSBs during 10 s using the × 60 oil objective. Fixed wavelength of ultraviolet A laser (405 nm) was used for laser microdissection in the temperature-controlled chamber with CO_2_ supplier. After laser treatment, cells were incubated at 37 °C for the indicated times. The intensity of each laser stripe in each time point was determined using confocal microscope. The kinetic analyses were performed using the NIS elements C software (Nikon). Each data series was normalized with respect to baseline values. Reconstitution assay has been carried out in U2OS cells transfected with TRAIP siRNA together with siRNA-resistant WT TRAIP, TRAIP-D2 or -D6 expression vector.

### Cell survival assay

HeLa cells in a 60-mm-diameter plate were transfected twice with the indicated siRNAs at 24-h intervals. Forty-eight hours after the second transfection, transfected cells were irradiated at the indicated doses using a radiation source. Fourteen days after irradiation, cells were washed with PBS, fixed, stained with 2% (w/v) methylene blue, and the colonies were counted. Reconstitution assay was carried out in HeLa cells transfected with siTRAIP together with siRNA-resistant WT TRAIP, TRAIP-D2, -D6 or -A6 expression vector.

### HR and non-homologous end joining assay

HR and non-homologous end joining assay were adapted from previous report^33^. Trypsinized 8 × 10^4^ U2OS DR-GFP or U2OS EJ5-GFP cells were reversely transfected with siRNA (with a final concentration of 25 nM) in a 12-well dish using Lipofectamine RNAiMAX (Invitrogen), as suggested by the manufacturer. Forty-eight hours later, the cells were transfected with 0.8 μg of I-SceI expression vector, or together with 0.4 μg of siRNA-resistant protein expression vector for reconstitution experiment, using Lipofectamine 2000 (Invitrogen). Seventy-two hours after I-SceI transfection, cells were trypsinized, and the percentage of GFP-positive cells was determined using flow cytometry.

### FokI assay

U2OS cells, containing the stably FokI restriction enzyme site, in 24-well plates were transfected with 0.4 μg of LacI-mCherry-FokI expression plasmid with 0.4 μg of GFP-fused TRAP WT, deletion mutant D6 or C-terminal region mutant A6. After 48 h, live cell imaging was performed with confocal microscopy.

### Tissue microarrays and immunohistochemistry

A lung adenocarcinoma tissue array (HLug-Ade150Sur-01) containing 75 cases of human lung adenocarcinoma patient tissue (64 adenocarcinomas, 7 bronchioloalveolar carcinomas, 3 mucoepidermoid carcinomas and 1 mucinous adenocarcinoma) and each matched normal adjacent tissue was obtained from US Biomax Inc. (Rockville, MD, USA). The classic method was used in immunohistochemistry to detect TRAIP and γH2AX^34^. Primary antibody against TRAIP (PA5-27699, Thermo Fisher Scientific) and γH2AX (ab11174, Abcam) were used at a concentration of 1:100 and 1:100, respectively. The staining intensity was assigned an arbitrary value, on a 4 scale (intensity score) as follows: non-stained (0), weak (1), moderate (2) and strong (3). The *H*-score was calculated by multiplying the intensity score and the fraction score (percentage of counted samples at each scale), producing the range 0–300, and repeated on three different areas. Cells with nuclear γH2AX foci on two to three different areas were counted and converted into percentages. Total counted cells in each sample were 70–100.

### Statistical analysis for immunohistochemistry

Statistical analysis was performed using the Prism 5 programme (GraphPad Software, San Diego, CA, USA). A Box-and-Whisker plot was used to compare the distribution of each sample in the groups. Comparison of TRAIP expression in the nucleus of tumour and adjacent non-tumour groups was analysed using *t*-test. Pearson correlation coefficient (*r*) analysis was used to compare TRAIP expression and γH2AX focus formation in the nucleus of tumour cells. A two-sided *P* value of less than 0.05 is considered to indicate a statistically significant result.

## Additional information

**How to cite this article:** Lee, N. S. *et al.* TRAIP/RNF206 is required for recruitment of RAP80 to sites of DNA damage. *Nat. Commun.* 7:10463 doi: 10.1038/ncomms10463 (2016).

## Supplementary Material

Supplementary Information Supplementary Figures 1-14 and Supplementary Table 1
