# Supplementary material for: TRAIP/RNF206 is required for recruitment of RAP80 to sites of DNA damage
Source: Nat Commun. 2016 Jan 19;7:10463. doi: 10.1038/ncomms10463 (PMC4735692; doi:10.1038/ncomms10463)
Supplement: Supplementary Information — Supplementary Figures 1-14 and Supplementary Table 1 [file ncomms10463-s1.pdf]

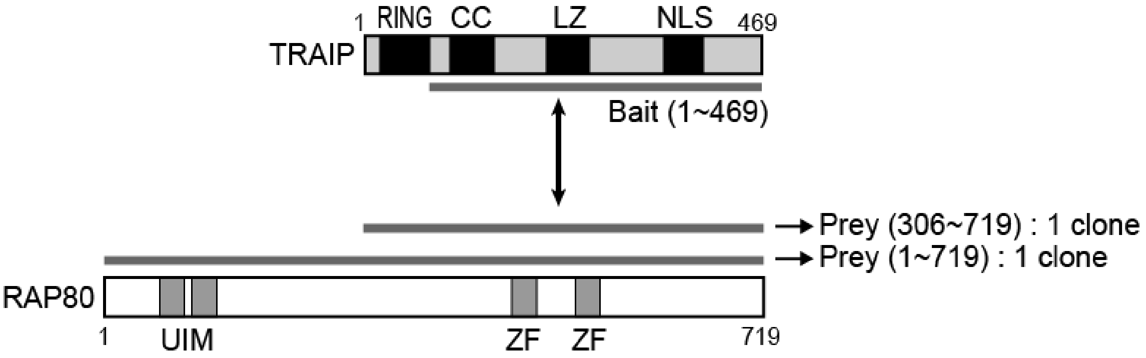

Supplementary Fig. 1. Schematic structure of TRAIP and RAP80. The prey line below TRAIP indicates bait and the two lines above RAP80 highlight the prey clones identified in the yeast two hybrid screen.

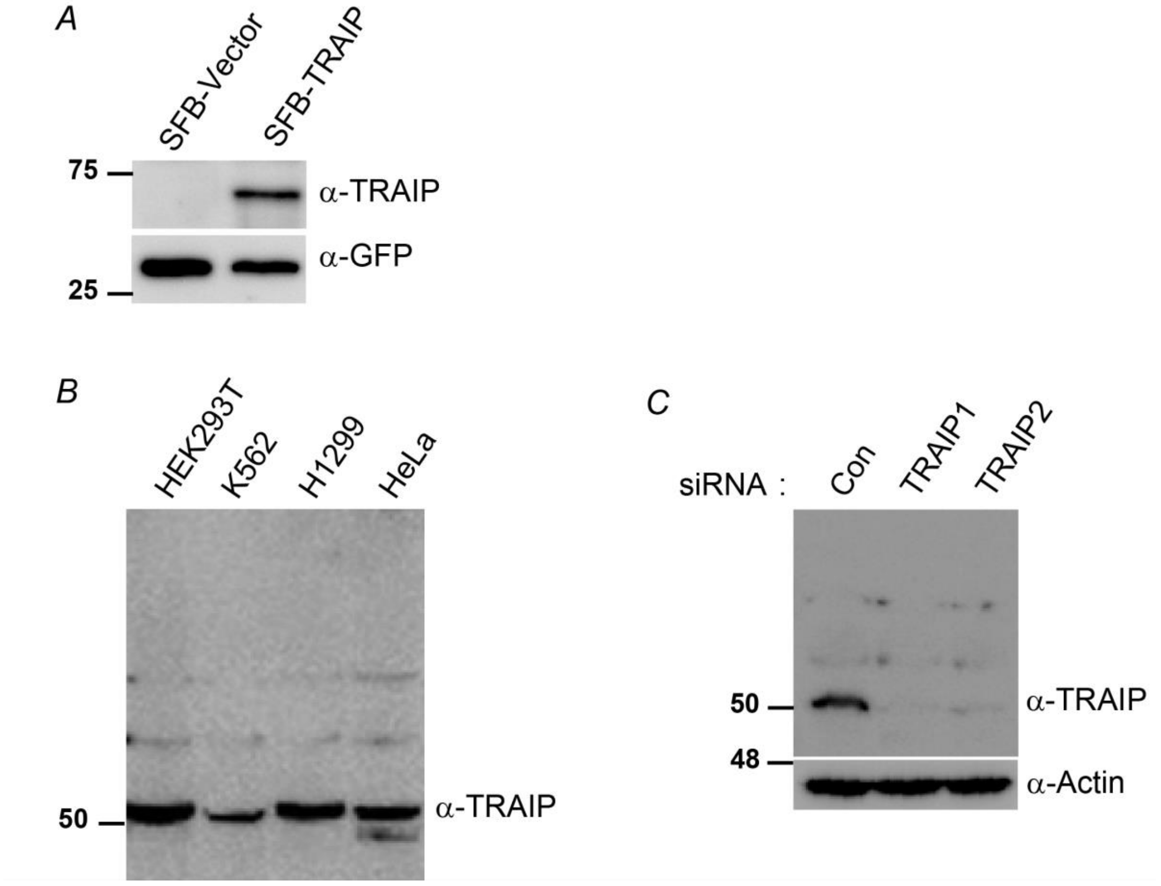

Supplementary Fig. 2. In-house TRAIP Antibody specificity. *(A)* In-house TRAIP antibody detects overexpressed TRAIP. SFB-Vector and SFB-TRAIP expression vector were transfected in HEK293T cells. GFP was used as an internal control for transfection. *(B)* Western blot analysis of TRAIP in HEK293T, K562, H1299, and HeLa cell lines using the in-house TRAIP antibody. The number indicates the molecular weight. *(C)* The in-house TRAIP antibody does not recognize TRAIP in cells with TRAIP depletion. See full blots in the Supplementary Fig. 14.

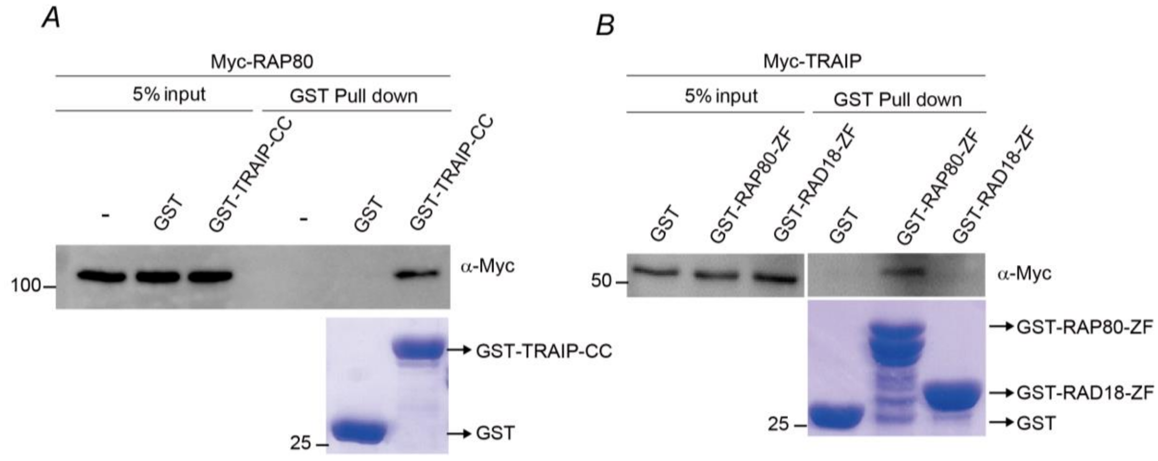

Supplementary Fig. 3. In *in vitro* binding assay of TRAIP and RAP80. *(A)* Cell lysates of 293T cells transfected with Myc-RAP80 expression plasmids were incubated with 2 μg of purified GST or GST-TRAIP-CC (coiled coil domain of TRAIP) recombinant protein for one hour at 4 °C. *(B)* Cell lysates of 293T cells transfected with Myc-TRAIP expression vector were incubated with 2 ug of purified GST or GST-RAP80-ZF (zinc finger domain of RAP80) recombinant protein for one hour at 4°C. Purified RAD18 zinc finger domain was used as a negative control. The bound complexes were separated by SDS-PAGE and subjected to Western blot analysis using the indicated antibody (*upper panel*). The Coomassie Blue-stained shows the purified GST fusion proteins (*lower panel*).

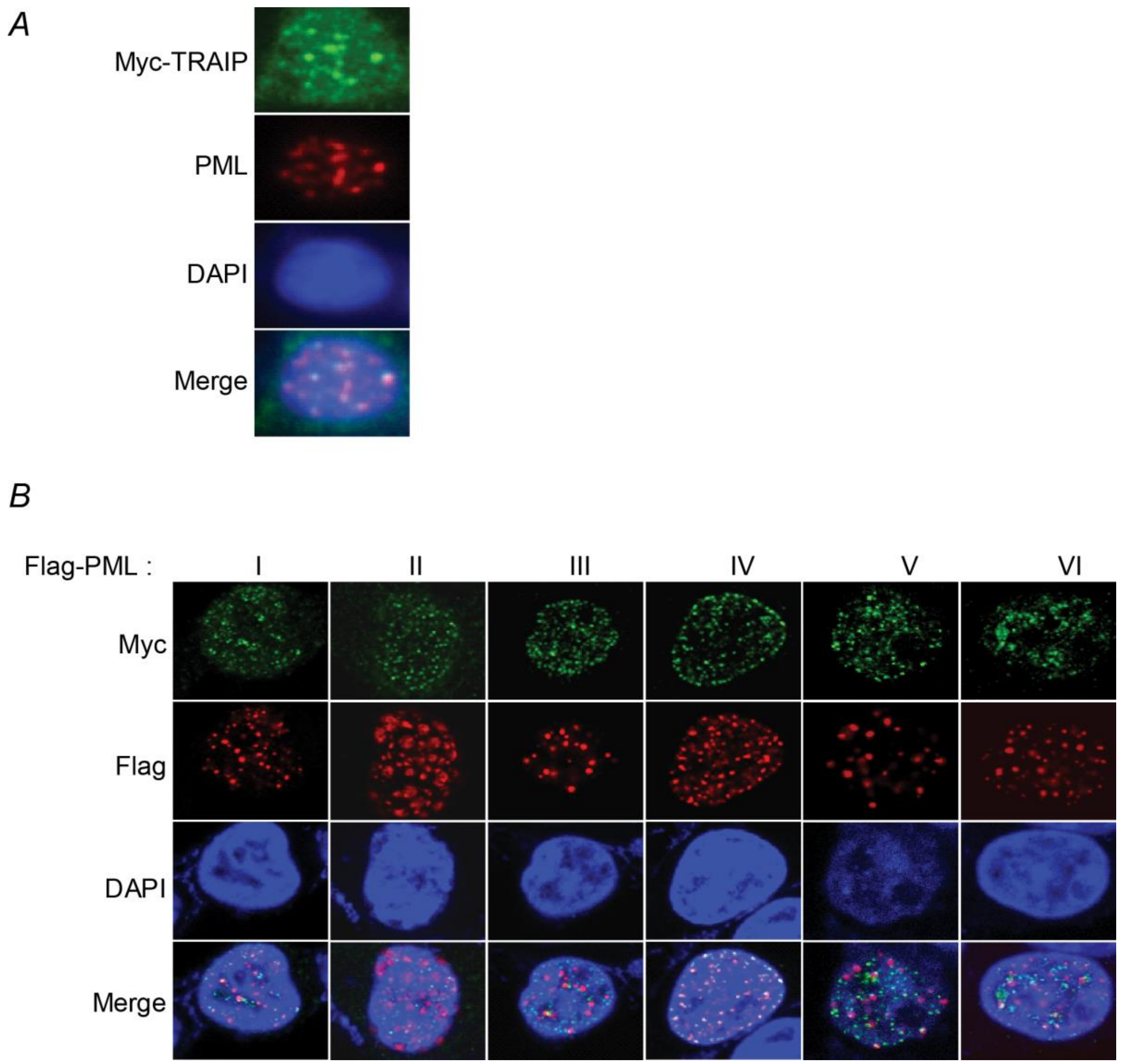

Supplementary Fig. 4. TRAIP foci were found in PML bodies in the absence of DNA damage (*A*) 293T cells were transfected with the Myc-TRAIP expression vector. After 24 hrs, transfected cells were fixed and immunofluorescence assays using anti-Myc and anti-PML antibodies. DAPI was used as an indicator for the nucleus. (*B*) 293T cells were transfected with indicated expression plasmid. After 24 hr, transfected cells were fixed and immunofluorescence assays using anti-Myc and -Flag antibodies. DAPI was used as an indicator for the nucleus.

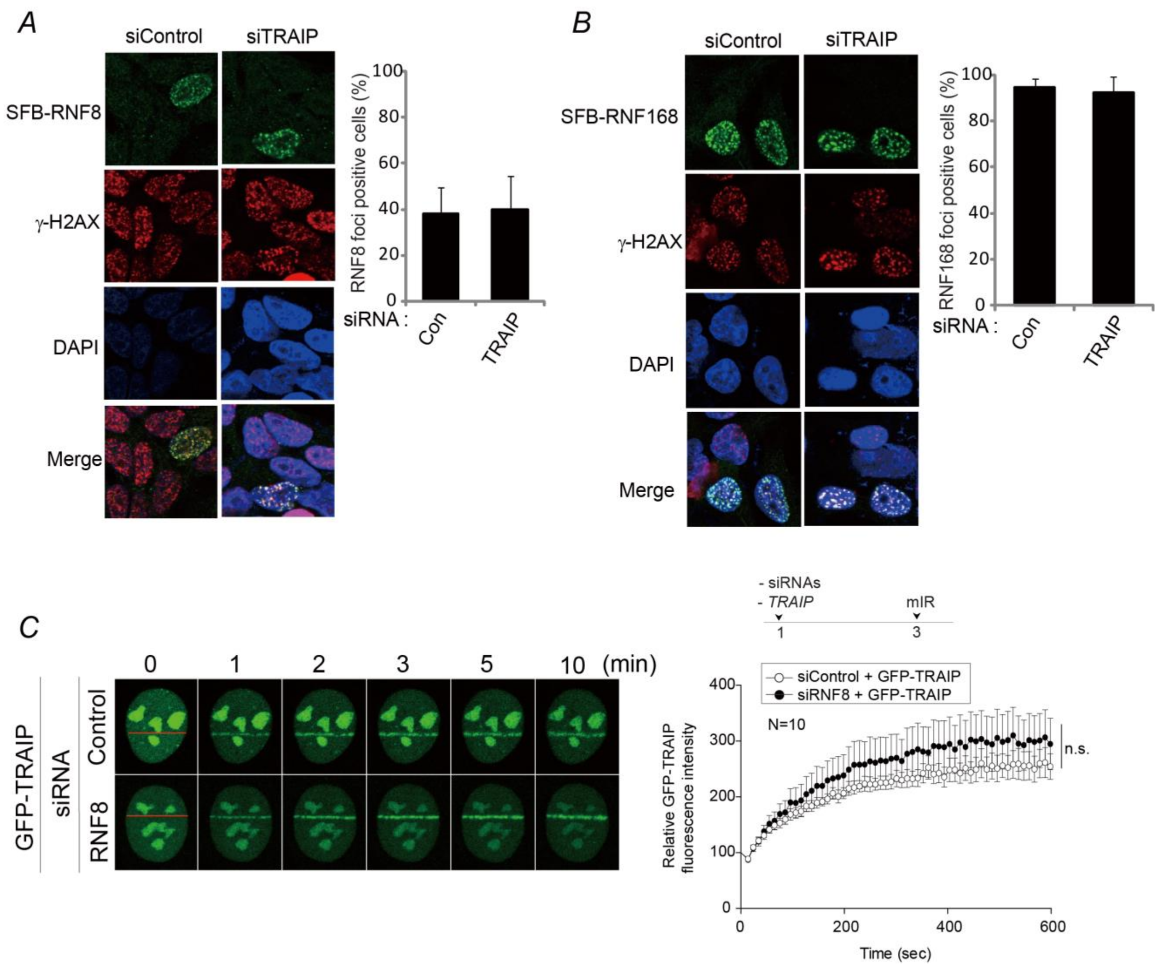

Supplementary Fig. 5. TRAIP is not involved in RNF8, RNF168-mediated DNA damage response. *(A and B)* SFB-RNF8 or SFB-RNF168 was transfected in 293T cells with TRAIP depletion. After 48 hrs, transfected 293T cells were exposed to 10 Gy of ionizing radiation. Four hour after irradiation, cells were fixed and stained with indicated antibodies. 4′,6-Diamidino-2-phenylindole (DAPI) was used as an indicator for the nucleus. The results represent the average of two independent experiments. Error bars indicate the standard deviation for each expression plasmid transfected cells. ***(C)*** GFP-TRAIP expression vector is transfected in U2OS cells with RNF8 depletion. After 48 hours incubation, the cells were subjected to laser microirradiation assay. Laser stripes were examined at the indicated times. Error bars indicate the standard deviation (N=10).

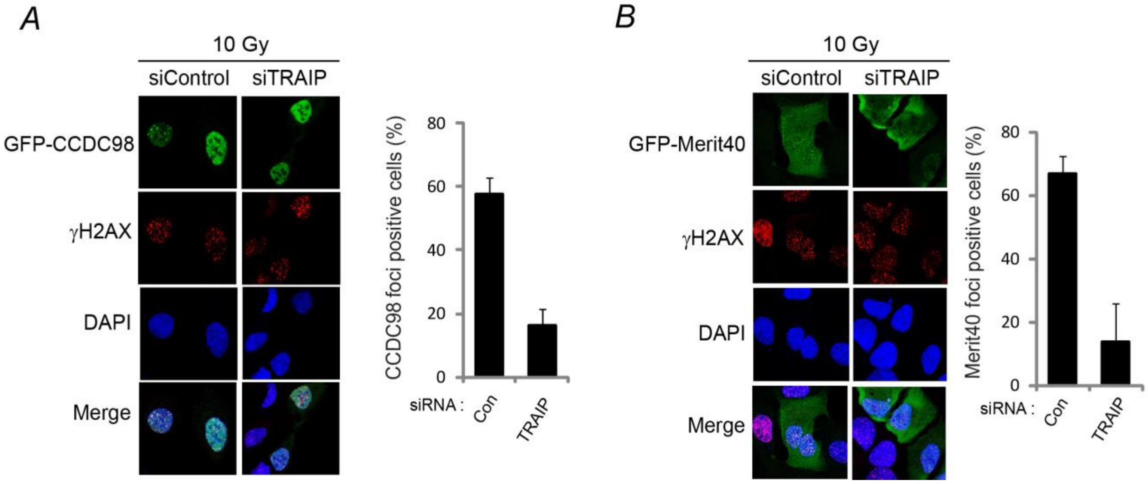

Supplementary Fig. 6. TRAIP is important for BRCA1-A complex recruitment to the sites of DNA lesions. GFP-CCDC98 *(A)* or GFP-Merit40 *(B)* was transfected in 293T cells depleted with TRAIP. After 48 hrs, transfected 293T cells were exposed to 10 Gy of ionizing radiation. Four hours after irradiation, cells were fixed and stained with γH2AX antibodies. DAPI was used as an indicator for the nucleus. The results represent the average of three independent experiments. Error bars indicate the standard deviation.

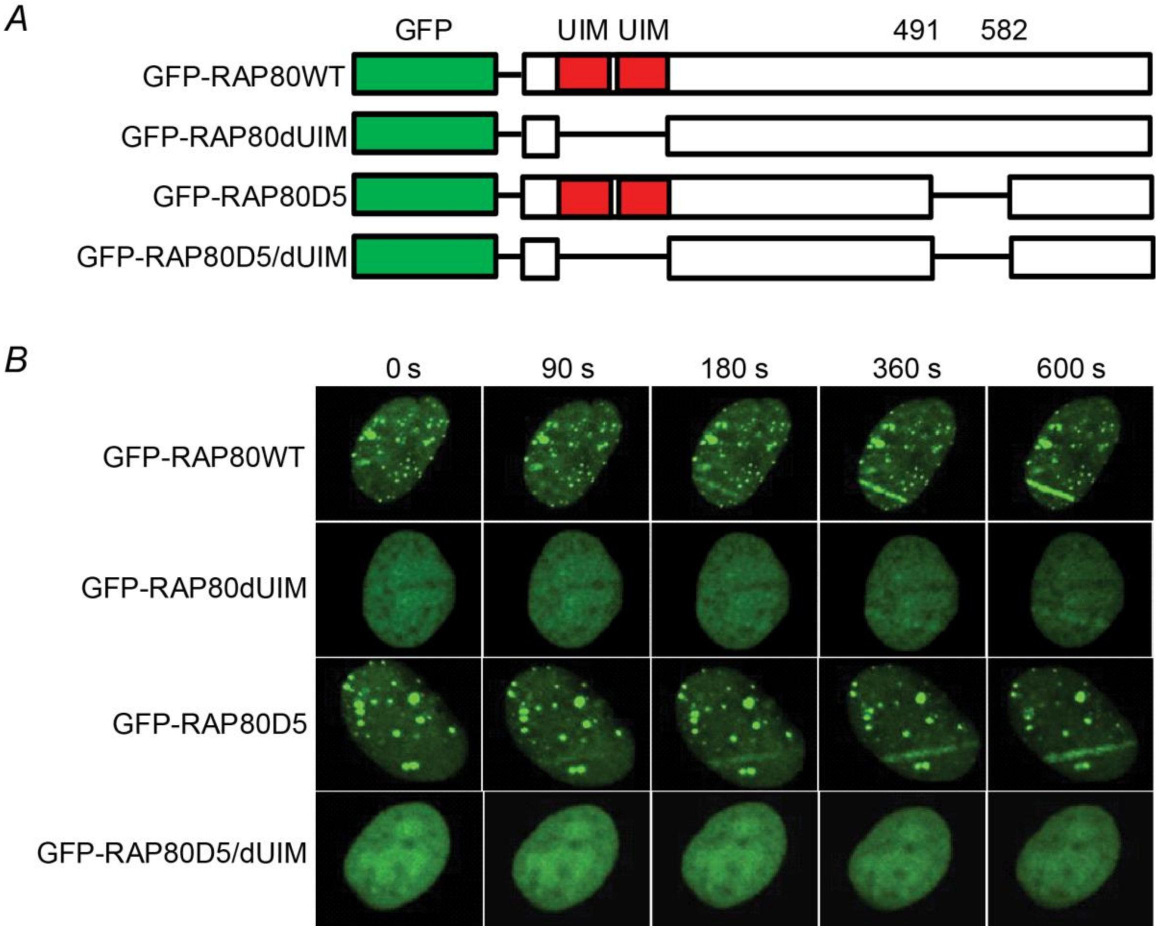

Supplementary Fig. 7. UIM and ZF domains of RAP80 are critical for RAP80 recruitment to the sites of DNA damage. *(A)* Diagram of GFP-fused RAP80 wild type and deletion mutants. *(B)* U2OS cells were transfected with GFP-tagged wild-type (WT) RAP80 or deletion mutant expression plasmids and then analyzed the kinetics of these proteins after laser microirradiation.

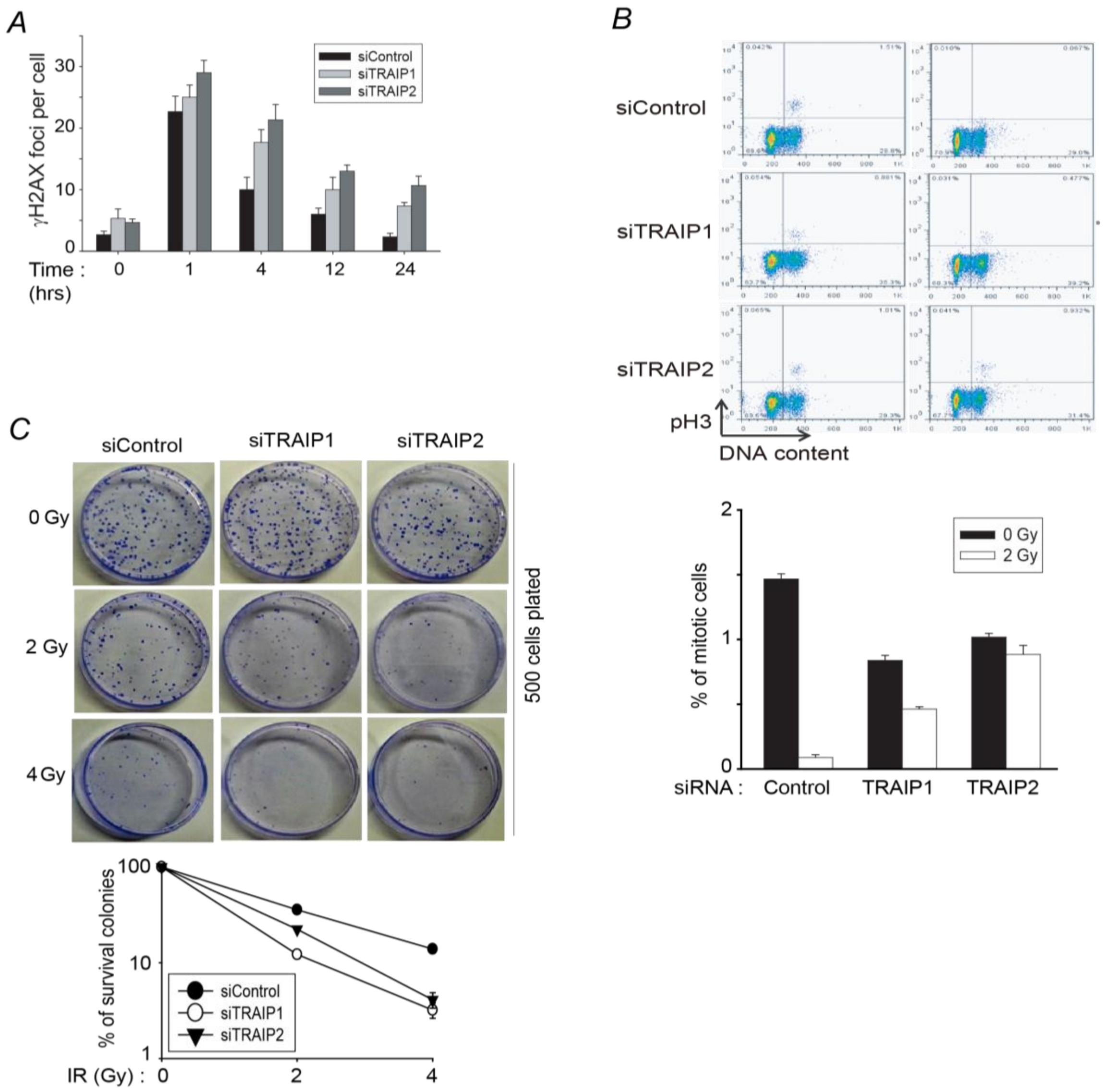

Supplementary Fig. 8. TRAIP is required for DNA damage response. *(A)* Counts of γH2AX foci at various time points after irradiation with 1 Gy of ionizing radiation in HeLa cells transfected with control or TRAIP siRNA1 or 2. The results represent the average of three independent experiments. *(B)* HeLa cells depleted with TRAIP were exposed to 0 or 2 Gy of IR. After one hour incubation, cells were fixed and subjected to staining with antibody to phosphorylated histone H3 (pH3) and propidium iodide. The percentages of mitotic cells were determined by FACS analysis. The boxed area in the top right panel indicates mitotic cells. (C) Radiation sensitivity of HeLa cells with TRAIP depletioin. HeLa cells were transfected with control or TRAIP siRNAs. Cells (N=500) were plated and irradiated with indicated doses of ionizing radiation. The number of surviving colonies was counted 14 days later. The error bar indicates standard deviation.

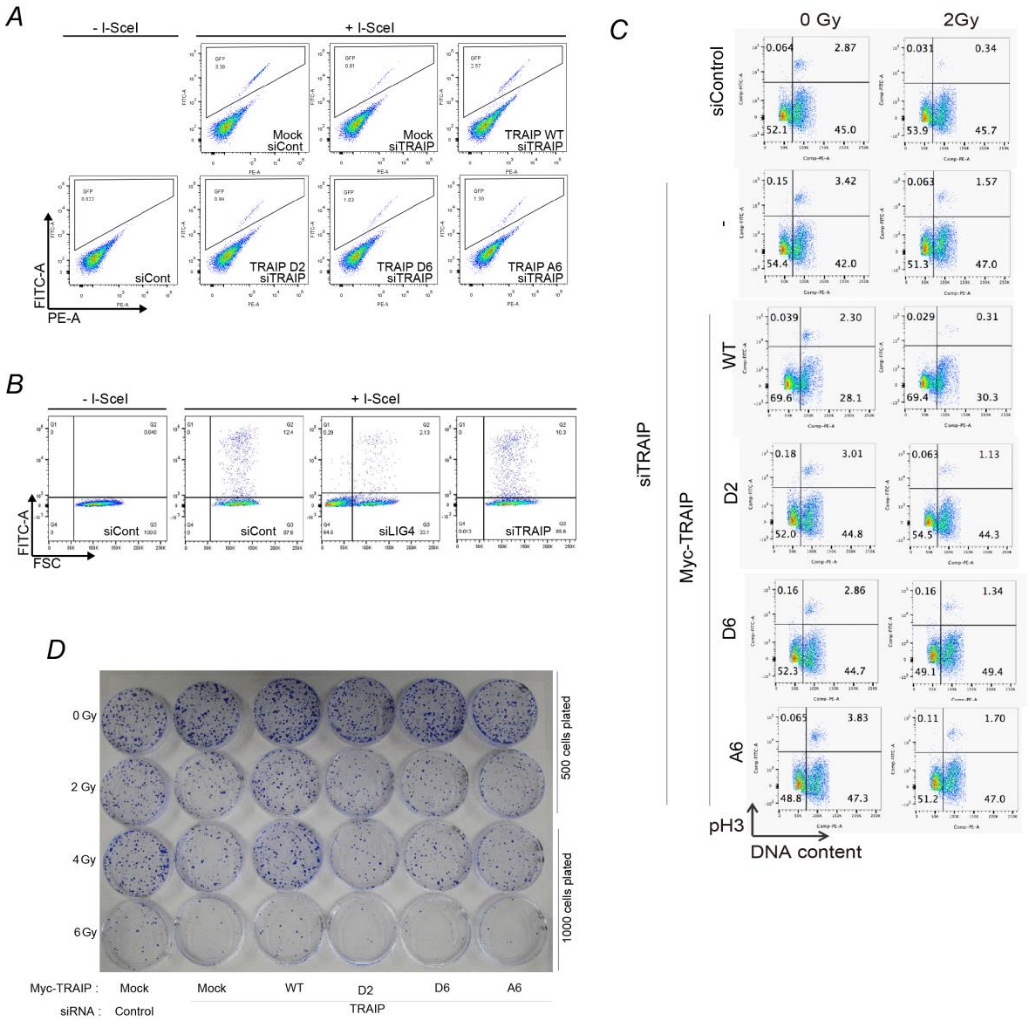

Supplementary Fig. 9. Complementation analysis with siRNA resistant TRAIP WT, TRAIP-D2, D6 or A6. *(A)* Representative FACS data for the figure 4B. DR-GFP U2OS cells were transfected with indicated siRNA resistant DNA. 24 hours later, I-SceI expression vector and the indicated siRNA were transfected. After 72 hours incubation, GFP positive cells were analyzed by FACS *(B)* Representative FACS data for the figure 4D. U2OS cells harboring the NHEJ reporter were transfected twice with indicated siRNA. After 48 hours of the first siRNA transfection, cells were transfected with I-SceI expression vector together with the second siRNA. 72 hours later, GFP positive cells were analyzed by FACS *(C)* Representative FACS data for the figure 4F. HeLa cells transfected with the combination of indicated siRNA and expression plasmid were exposed to 0 or 2 Gy of IR. Cells were incubated for 1 hr and then fixed and subjected to staining with antibody to phosphorylated histone H3 (pH3) and propidium iodide. The percentages of mitotic cells were determined by fluorescence-activated cell sorting analysis. The boxed area in the top right panel indicates mitotic cells. *(D)* Clonogenic IR sensitive data for the figure 4G. HeLa cells were transfected with combination of indicated siRNA and expression plasmid. Cells (N=500 or N=1000) were plated and irradiated with indicated doses of ionizing radiation. The number of surviving colonies was counted 14 days later.

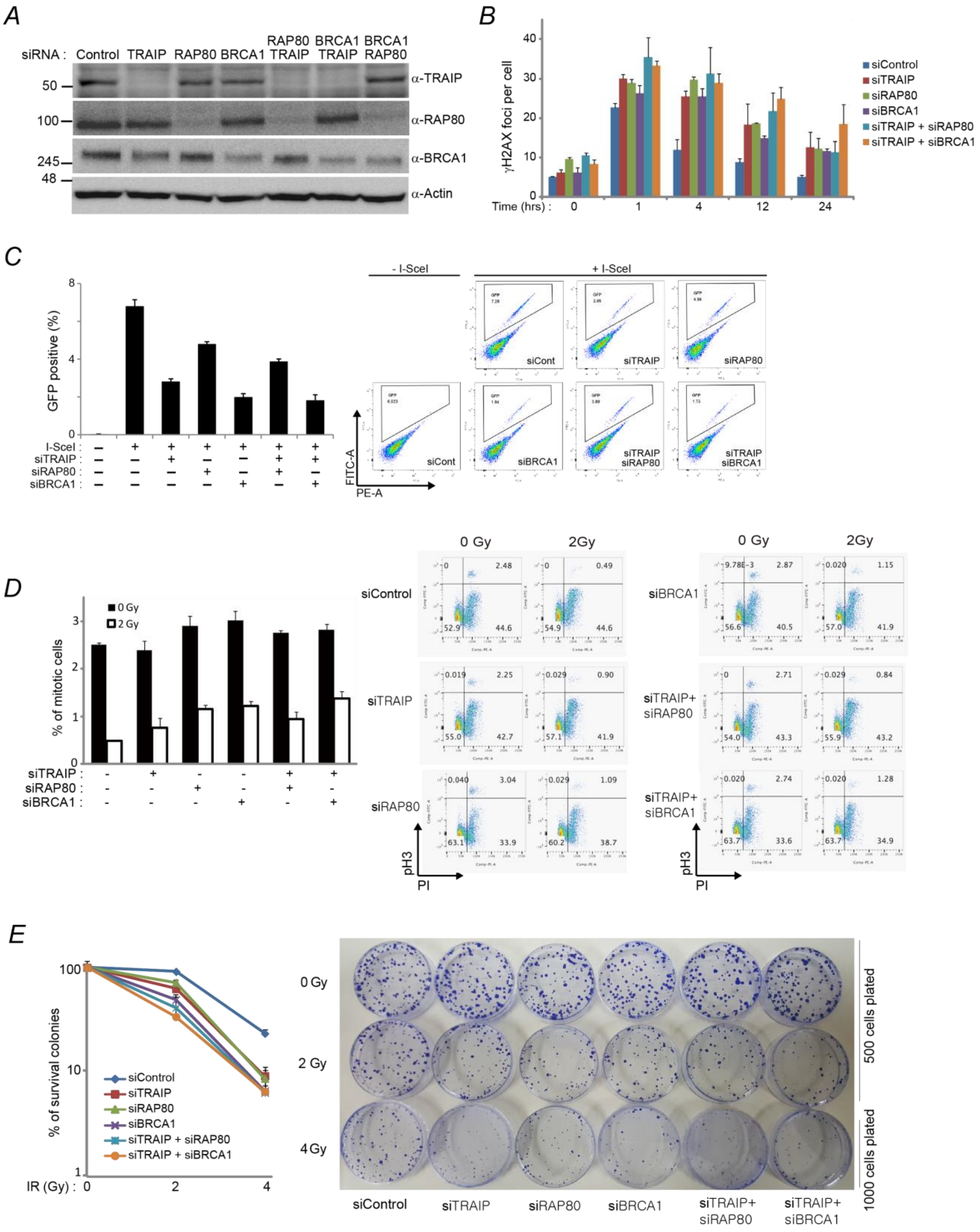

Supplementary Fig. 10. Genetic interaction between TRAIP and RAP80 or BRCA1
(A) Transfection of individual or combination of siRNAs effectively downregulated expression of target proteins. Indicated siRNAs were transfected in 293T cells. After 48 hrs, cell lysates were subjected to Western blotting analysis. *(B)* Counts of γH2AX foci at various time points after irradiation with 1 Gy of ionizing radiation in HeLa cells transfected with individual or combination of indicated siRNAs. *(C)* Comparison of

homologous recombination repair capacity in U2OS cells transfected with indicated siRNAs (left panel). Representative Flow-cytometry data (right panel). *(D)* Comparison of checkpoint defects in HeLa cells transfected with individual or combination of siRNAs (left panel). Representative Flow-cytometry data (right panel). *(E)* Comparison of radiation sensitivity. HeLa cells were transfected with individual or combination of indicated siRNA. Cells (N=500 or N=1000) were plated and irradiated with indicated doses of ionizing radiation. The number of surviving colonies was counted 14 days later. See full blots in the Supplementary Fig. 14.

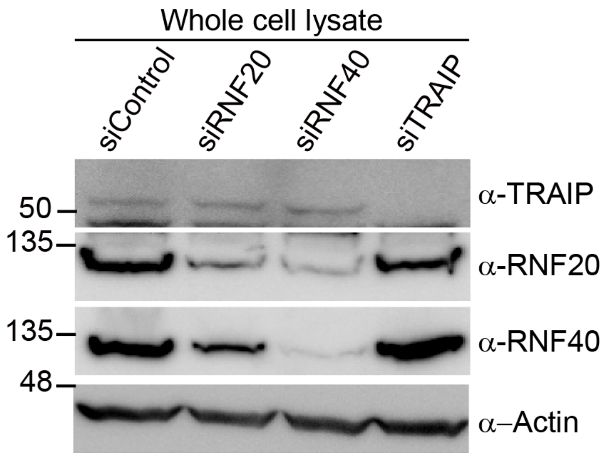

Supplementary Fig. 11. Western blot analysis for TRAIP, RNF20 or RNF40 expression in HeLa cells transfected with the control, TRAIP, RNF20 or RNF40 siRNA. The indicated siRNAs were transfected into HeLa cells. After 48 hrs, transfected cell lysates were subjected to Western blotting analysis using the indicated antibodies. See full blots in the Supplementary Fig. 14.

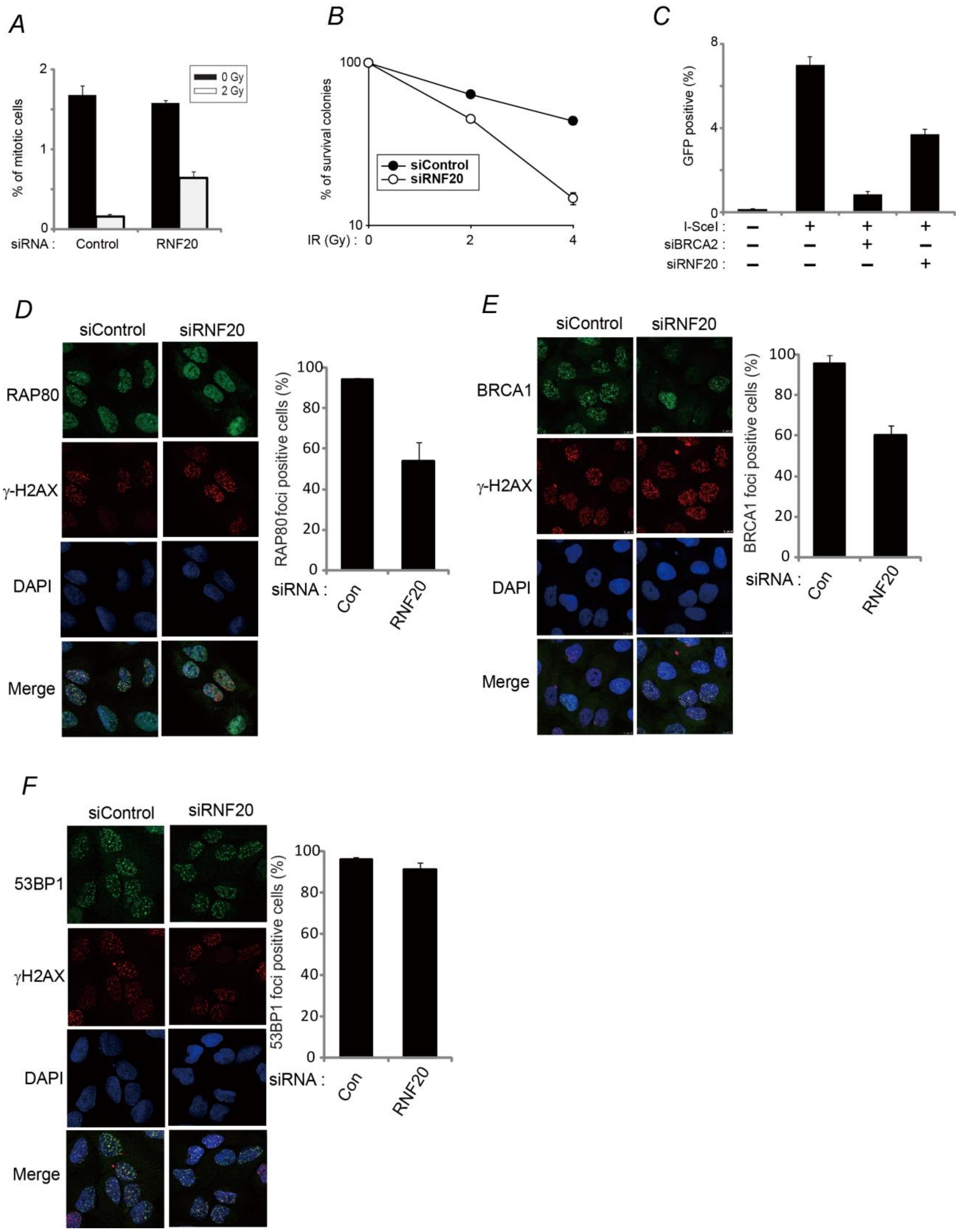

Supplementary Fig. 12. The functional analysis of RNF20 on DNA damage response pathway. *(A)* Depletion of RNF20 leads to checkpoint defects. HeLa cells transfected with indicated siRNA were exposed to 0 or 2 Gy of ionizing radiation. Cells were incubated for 1 hr and then fixed and subjected to staining with antibody to phosphorylated histone H3 (pH3) and propidium iodide. The percentages of mitotic cells

were determined by fluorescence-activated cell sorting analysis. The results represent the average of two independent experiments. Error bars indicate the standard deviation. *(B)* Depletion of RNF20 leads to radiation sensitivity. HeLa cells were transfected with control or RNF20 siRNAs. 48 hours after transfection, 500 cells were plated and irradiated with indicated doses of ionizing radiation. The number of surviving colonies was counted 14 days later. These experiments were performed in triplicate, and the results represent the average of three independent experiments. *(C)* Depletion of RNF20 reduced the homologous recombination. DR-GFP U2OS cells were transfected with indicated siRNA. 48 hours after transfection, I-SceI expression vector was transfected. 3 days later, GFP positive cells were analyzed by flowcytometry. *(D-F)* 293T cells were transfected with control or RNF20 siRNAs. After 48 hr transfected 293T cells were exposed to 10 Gy of ionizing radiation. Four hours after irradiation, cells were fixed and stained with anti-RAP80, -BRCA1, -53BP1or -γH2AX antibodies. DAPI was used as an indicator for the nucleus. The results represent the average of three independent experiments. Error bars indicate the standard deviation.

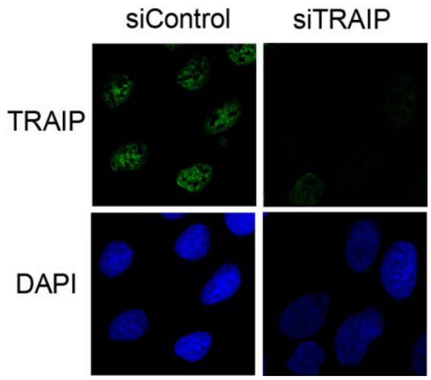

Supplementary Fig. 13. TRAIP antibody specificity for immunofluorescent assay. HeLa cells were transfected with indicated siRNA. After 48 hours, transfected HeLa cells were fixed and stained with anti-TRAIP antibody. DAPI was used as an indicator for the nucleus.

Figure 1C

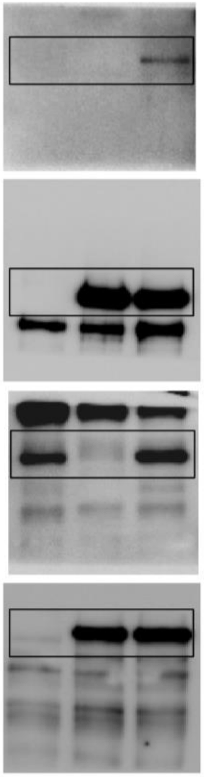

Figure 1D

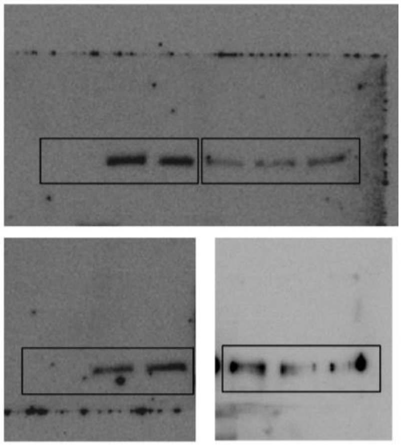

Figure 3D

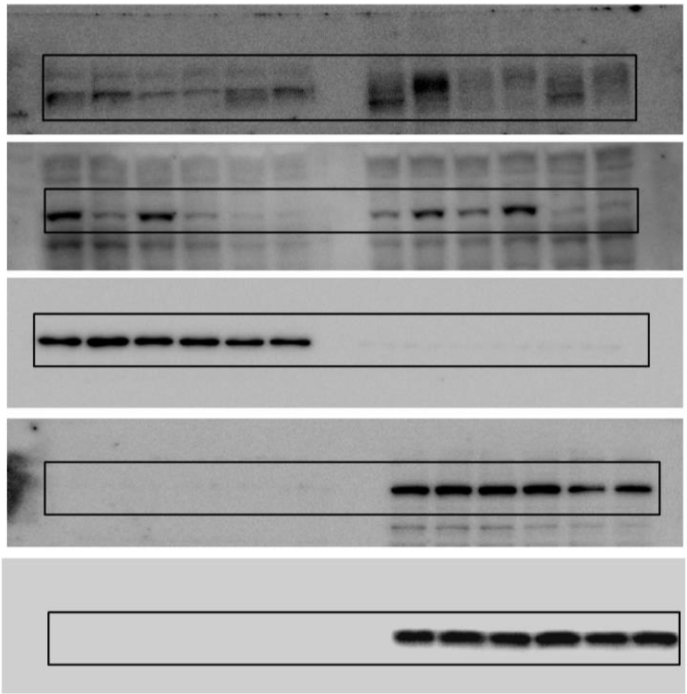

Figure 3E

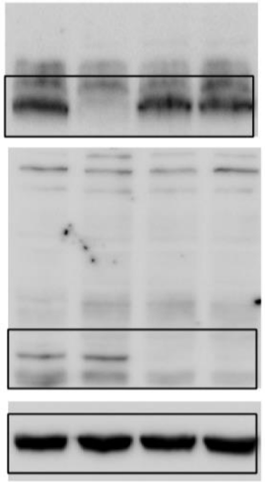

Figure 5C

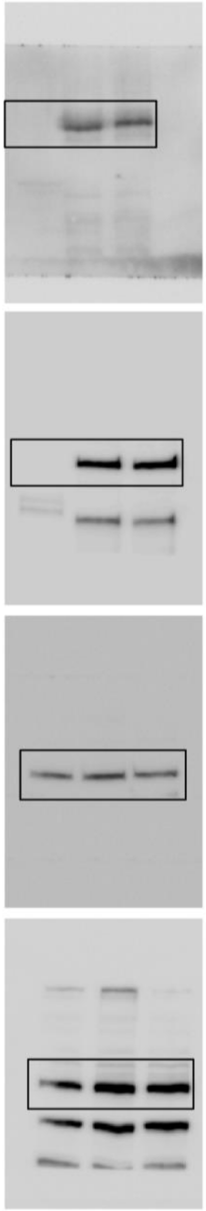

Figure 5D

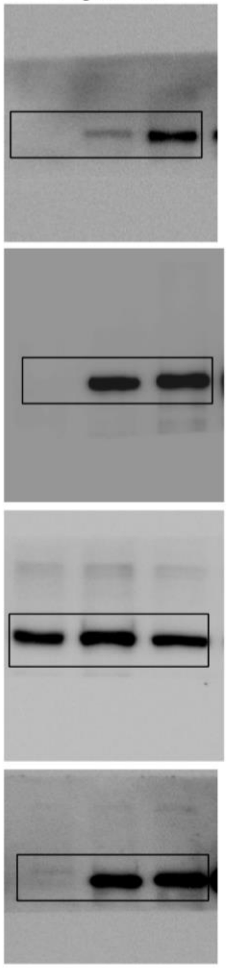

Figure 5G

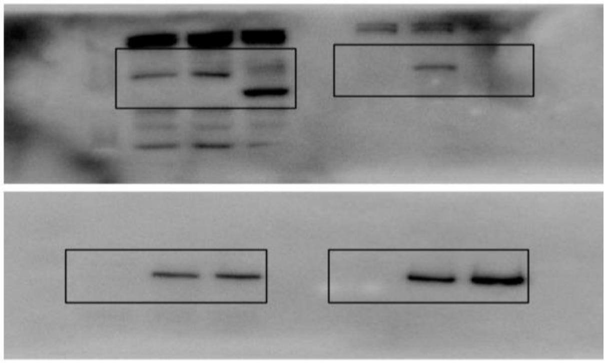

Figure 5I

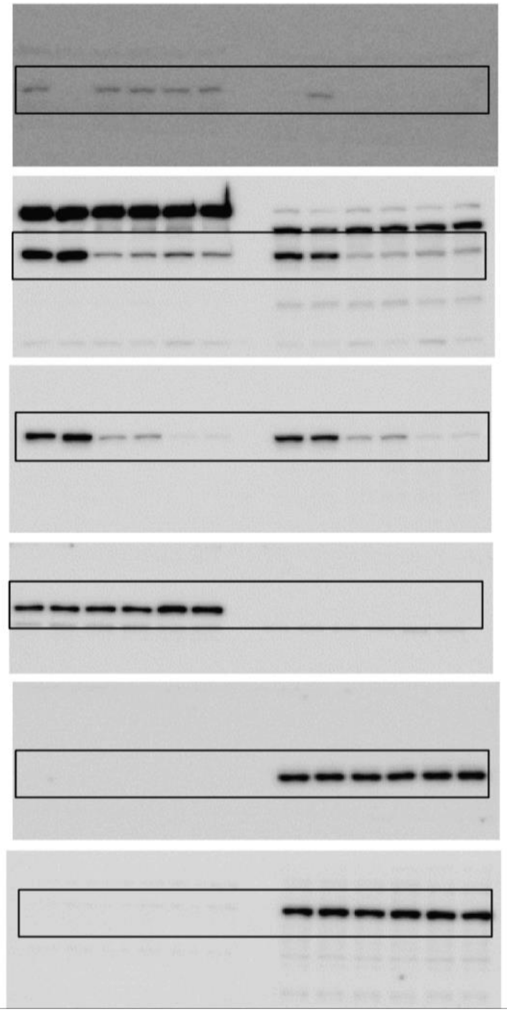

Supplementary Figure 2A

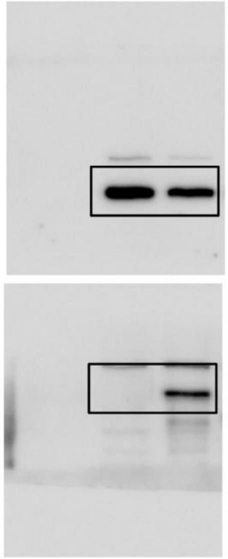

Supplementary Figure 10A

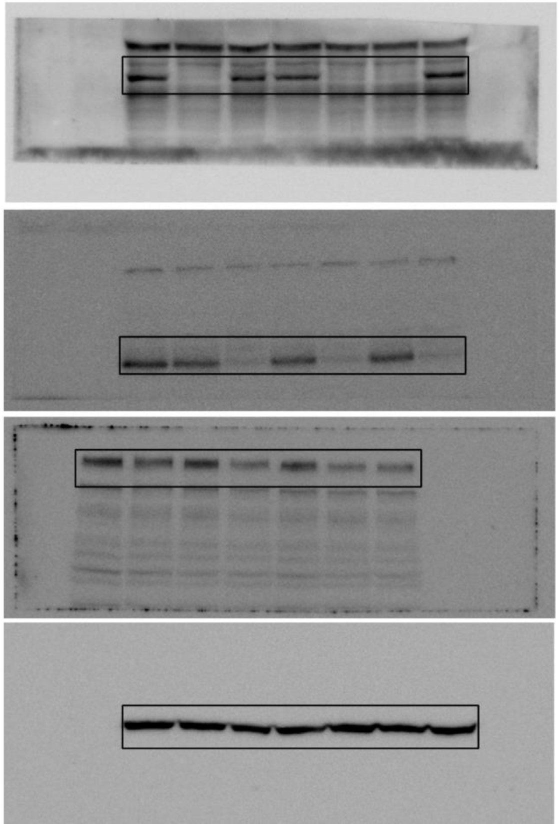

Supplementary Figure 11

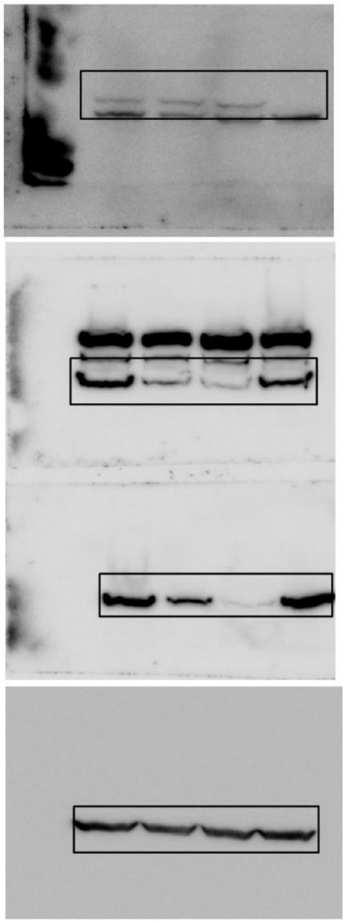

Supplementary Fig. 14. Uncropped Western blots shown in figures and supplementary figures.

Supplementary table 1. The list of 37 positive clones with the highest-galactosidase activity from the yeast two-hybrid screen with the full-length RAP80 as bait and a human HeLa cDNA library as prey. Clones containing the TRAIP cDNA were denoted by red font.

Prey ID	Description
**AD-Hybrid - 1,22**	The activation domain (AD) is fused in frame to the 3rd aa of ubiquitin-conjugating enzyme E2I (**UBE2I**), transcript variant 1 (NM_003345).
**AD-Hybrid - 2**	The activation domain (AD) is fused in frame to the 68th aa of complement component 1, q subcomponent binding protein (**C1QBP**), nuclear gene encoding mitochondrial protein (NM_001212).
**AD-Hybrid - 3**	The activation domain (AD) is fused in frame to the 123rd aa of coiled-coil alpha-helical rod protein 1 (**CCHCR1**), transcript variant 1 (NM_001105564).
**AD-Hybrid - 4**	The activation domain (AD) is fused in frame to the 205th aa of coiled-coil alpha-helical rod protein 1 (**CCHCR1**), transcript variant 1 (NM_001105564).
**AD-Hybrid - 5**	The activation domain (AD) is fused to 5' UTR (untranslated region) of complement component 1, q subcomponent binding protein (**C1QBP**), nuclear gene encoding mitochondrial protein mRNA at -24 nt (NM_001212).
**AD hybrid - 6**	The activation domain (AD) is fused to 5' UTR of TRAF interacting protein (**TRAIP**) mRNA at -86 nt (NM_005879).
**AD hybrid - 7**	The activation domain (AD) is fused to 5' UTR of SMT3 suppressor of mif two 3 homolog 1 (S. cerevisiae) (**SUMO1**), transcript variant 1 mRNA at -122 nt (NM_003352).
**AD hybrid - 8**	The activation domain (AD) is fused to 5' UTR of cleavage and polyadenylation specific factor 6, 68kDa (**CPSF6**) mRNA at -5 nt (NM_007007).
**AD hybrid - 9**	The activation domain (AD) is fused in frame to the 3rd aa of cleavage and polyadenylation specific factor 6, 68kDa (**CPSF6**) (NM_007007).
**AD-Hybrid - 10**	The activation domain (AD) is fused in frame to the 21st aa of cleavage and polyadenylation specific factor 6, 68kDa (**CPSF6**) (NM_007007).
**AD-Hybrid - 11**	The activation domain (AD) is fused in frame to the 13th aa of complement component 1, q subcomponent binding protein (**C1QBP**), nuclear gene encoding mitochondrial protein (NM_001212).
**AD-Hybrid - 12**	The activation domain (AD) is fused in frame to the 1547th aa of caspase 8 associated protein 2 (**CASP8AP2**), transcript variant 2 (NM_001137667).
**AD-Hybrid - 13**	The activation domain (AD) is fused in frame to the 69th aa of complement component 1, q subcomponent binding protein (**C1QBP**), nuclear gene encoding mitochondrial protein (NM_001212).
**AD-Hybrid - 14**	The activation domain (AD) is fused in frame to the 30th aa of complement component 1, q subcomponent binding protein (**C1QBP**), nuclear gene encoding mitochondrial protein (NM_001212).
**AD-Hybrid - 15**	The activation domain (AD) is fused to 5' UTR of SMT3 suppressor of mif two 3 homolog 1 (S. cerevisiae) (**SUMO1**), transcript variant 1 mRNA at -92 nt (NM_003352).
**AD hybrid - 16**	The activation domain (AD) is fused to 5' UTR of ubiquitin-conjugating enzyme E2I (**UBE2I**), transcript variant 4 mRNA at -84 nt (NM_194261).
**AD hybrid - 17**	The activation domain (AD) is fused in frame to the 70th aa of TRAF interacting protein (**TRAIP**) (NM_005879).
**AD hybrid - 18-21**	The activation domain (AD) is fused to cleavage and polyadenylation specific factor 6, 68kDa (CPSF6) (NM_007007).

**AD hybrid - 23**	The activation domain (AD) is fused to ubiquitin-conjugating enzyme E2I (UBE2I), transcript variant 1 (NM_003345).
**AD-Hybrid - 24**	The activation domain (AD) is fused out of frame to the 131st aa of mucin 1, cell surface associated (**MUC1**), transcript variant 9 (NM_001204285).
**AD-Hybrid - 25**	The activation domain (AD) is fused in frame to the 3rd aa of protein inhibitor of activated STAT, 1 (**PIAS1**) (NM_016166).
**AD-Hybrid - 26**	The activation domain (AD) is fused to 5' UTR of complement component 1, q subcomponent binding protein (**C1QBP**), nuclear gene encoding mitochondrial protein mRNA at -6 nt (NM_001212).
**AD hybrid - 27-32**	The activation domain (AD) is fused to complement component 1, q subcomponent binding protein (C1QBP), nuclear gene encoding mitochondrial protein (NM_001212).
**AD hybrid - 33**	The activation domain (AD) is fused in frame to the 41st aa of lactate dehydrogenase A (**LDHA**), transcript variant 3 (NM_001165414).
**AD hybrid - 34**	The activation domain (AD) is fused in frame to the 46th aa of TNF receptor-associated factor 3 (**TRAF3**), transcript variant 3 (NM_003300).
**AD hybrid - 35**	The activation domain (AD) is fused to 5' UTR of TRAF interacting protein (**TRAIP**) mRNA at -20 nt (NM_005879).
**AD hybrid - 36**	The activation domain (AD) is fused to TRAF interacting protein (TRAIP) (NM_005879).
**AD-Hybrid - 37**	The activation domain (AD) is fused in frame to the 9th aa of GTF2I repeat domain containing 1 (**GTF2IRD1**), transcript variant 1 (NM_016328).
